# Detection and Complete Genome Analysis of Porcine Circovirus 2 (PCV2) and an Unclassified CRESS DNA Virus from Diarrheic Pigs in the Dominican Republic: First Evidence for Predominance of PCV2d from the Caribbean Region

**DOI:** 10.3390/v14081799

**Published:** 2022-08-17

**Authors:** Kerry Gainor, Yussaira Castillo Fortuna, Angeline Steny Alakkaparambil, Wendy González, Yashpal Singh Malik, Souvik Ghosh

**Affiliations:** 1 Department of Biomedical Sciences, Ross University School of Veterinary Medicine, Basseterre P.O. Box 334, Saint Kitts and Nevis; 2 Department of Biotechnology, School of Bio Sciences and Technology, Vellore Institute of Technology, Vellore 632014, India; 3 Epidemiological Surveillance Division, Dirección General de Ganadería, Santo Domingo 10410, Dominican Republic; 4 School of Veterinary Medicine, Faculty of Agronomic and Veterinary Sciences, Autonomous University of Santo Domingo, Calle Camino de Engombe 10904, Dominican Republic; 5 College of Animal Biotechnology, Guru Angad Dev Veterinary and Animal Science University, Ludhiana 141012, India

**Keywords:** complete genome analysis, Dominican Republic, genotype shift, porcine circovirus 2, porcine circovirus 2d, porcine-associated unclassified CRESS DNA virus

## Abstract

We report here high rates (47.5%, 48/101) of detection of porcine circovirus 2 (PCV2) in diarrheic pigs from three pig farms in the Dominican Republic. Seventeen of the PCV2 positive samples, representing the three pig farms, different age groups and sampling periods (2020–2021), were amplified for the complete PCV2 genome. Based on analysis of open reading frame 2 and complete genome sequences, the 17 PCV2 strains were assigned to the PCV2d genotype. Significant differences were observed in PCV2 detection rates between the vaccinated (20% (10/50)) and unvaccinated (62.5% (10/16) and 80% (28/35)) farms, corroborating previous observations that PCV2a-based vaccines confer protection against heterologous PCV2 genotypes. The present study is the first to report detection and molecular characterization of PCV2 from the Dominican Republic, warranting large-scale molecular epidemiological studies on PCV2 in pig farms and backyard systems across the country. For the first time, PCV2d was identified as the predominant PCV2 genotype in a study from the Caribbean region, suggesting that a genotype shift from PCV2b to PCV2d might be happening in the Caribbean region, which mirrored the current PCV2 genotype scenario in many other parts of the world. Besides PCV2, we also identified a pigeon circovirus-like virus, and a circular Replication-associated protein (Rep)-encoding single-stranded (CRESS) DNA virus, which was characterized for the complete genome. The CRESS DNA virus shared a similar genomic organization and was related to unclassified CRESSV2 DNA viruses (belonging to the Order *Cirlivirales*) from porcine feces in Hungary, indicating that related unclassified CRESS DNA viruses are circulating among pigs in different geographical regions, warranting further studies on the epidemiology and biology of these novel viruses.

## 1. Introduction

Porcine circoviruses (PCV), members of the genus *Circovirus* within family *Circoviridae*, are small non-enveloped viruses with a circular, single-stranded ambisense DNA genome [1,2]. To date, at least four species of PCV (designated as *porcine circovirus 1–4*) have been recognized in pigs [2]. Among them, PCV2 is ubiquitous in swine populations worldwide, incurring significant economic losses to the pork industry [2,3,4,5]. In pigs, PCV2 has been associated with various clinical and subclinical conditions (post-weaning multisystemic wasting syndrome (PMWS)/PCV2 systemic disease, porcine dermatitis, and nephropathy syndrome (PDNS), PCV2-associated pneumonia, reproductive disorders, and enteric diseases) that are collectively referred to as porcine circovirus-associated diseases (PCVAD) [5,6,7]. Besides circoviruses, several diverse circular replication-associated protein (Rep)-encoding single-stranded DNA (CRESS DNA) viral sequences have been reported from healthy and clinically ill pigs, although the true host/s and pathogenesis of these viruses are largely unknown [8,9,10].

The PCV2 genome consists of two major open reading frames (ORF): ORF1 codes for the replication-related proteins—Rep and Rep’, whilst ORF2 encodes the capsid protein (Cap) [1,11,12]. The PCV2 Cap plays a crucial role in viral attachment to host cells, and represents the major immunogenic protein, forming the basis of the current PCV2 vaccines [5,11,13,14]. Based on differences in ORF2 sequences, PCV2 strains have been classified into at least eight established genotypes (PCV2a-h) [2,5,14,15,16,17]. A ninth PCV2 genotype, PCV2i, has also been proposed in a study from the US [16]. During the mid-2000s, PCV2b replaced PCV2a as the predominant global genotype, and more recently, PCV2d has emerged as the major genotype in many parts of the world [2,5,14,15,16,17]. Although the currently licensed PCV2 vaccines, based on PCV2a, appear to confer cross protection against the other PCV2 genotypes, the implications of the two major PCV2 genotype shifts, especially the emergence of PCV2d, on vaccine efficacy and disease severity in porcine populations remains to be clearly elucidated [5,14,15,16,18,19,20,21,22,23,24,25,26,27,28,29,30,31].

Although pig farming constitutes an important component of the livestock economy in the Caribbean region [32], limited information is available on PCV2 infection in pigs from the region so far [32,33,34,35,36,37,38]. Single reports from the Dominican Republic, Haiti, and Trinidad and Tobago have documented high prevalence of antibodies to PCV2 in pig farms/premises [32,33,34,35]. On the other hand, two molecular epidemiological studies from Cuba and a single study from St. Kitts identified PCV2b as the major PCV2 genotype [36,37,38]. In the present study, based on diarrheic fecal samples collected during 2020–2021, we report for the first time high rates of detection and complete genomic analysis of PCV2 in pigs from the Dominican Republic, and provide the first evidence for predominance of PCV2d from a Caribbean country. Furthermore, we also identified a pigeon circovirus-like viral sequence, and an unclassified CRESS DNA virus, which was analyzed for the complete genome.

## 2. Materials and Methods

### 2.1. Sampling

The present study was based on 101 fecal samples that were collected from pigs with diarrhea at three different farms in the Dominican Republic for a project on ‘detection and molecular characterization of adenovirus (AdV) and *Rotavirus A* (RVA) in porcine enteritis’ (Supplementary Material S1). During August–November 2020, 35 fecal samples were obtained from a pig farm (housing approximately 300 pigs) in the municipality of Cabrera, whilst 16 fecal samples were collected from a pig farm (approximately 150 pigs) in the municipality of Pedro Brand. Between January and February 2021, 50 fecal samples were collected on a pig farm (approximately 500 pigs) in the municipality of Villa Mella. The maximum number of diarrheic samples were from weaners (34.65%, 35/101 animals), followed by growers (17.82%, 18/101) and piglets (16.83%, 17/101). Most of the diarrheic weaners and growers exhibited retarded growth, pallor, and poor gain in body weight. Four of the diarrheic pigs died (one each of a piglet, weaner, grower, and dry sow).

The diarrheic pigs were identified and closely monitored by the farm veterinarian. When the animal defecated, a sterile container (4 oz. Specimen Cup, Dynarex Corporation, New York, NY, USA) was held near the rectal orifice to collect a projectile of the liquid/semi-liquid feces. To avoid contamination, the veterinarian changed gloves and personal protective equipment between collection of samples. After sampling, the container was sealed with sterile tape, placed in two layers of biohazard bags, and transported under cold chain to the laboratory. The samples were stored at −20 °C until further use. The Institutional Animal Care and Use Committee (IACUC) of the Ross University School of Veterinary Medicine (RUSVM), St. Kitts and Nevis, acknowledged the collection and use of the porcine samples for the present study (RUSVM IACUC #: TSU6.10.22).

### 2.2. Amplification of Viral Genome

Viral DNA was extracted from the porcine fecal samples using the QIAamp Fast DNA Stool Mini Kit (Qiagen Sciences, Germantown, MD, USA) following the manufacturer’s instructions. The samples were screened for PCV2 DNA by a PCV2-specific nested PCR assay targeting the Rep-encoding gene (Supplementary Material S2). The complete genomes of PCV2 strains were amplified using three overlapping nested PCRs that included the screening PCR assay (Supplementary Material S2). Fecal samples from Cabrera and Pedro Brand were screened for novel circoviruses/other CRESS DNA viruses by a circovirus/cyclovirus pan-*rep* nested PCR assay as described in previous studies [39,40]. The full-length genome of the porcine-associated CRESS DNA virus (designated as CRESSV2/ENG22) was amplified by an inverse nested PCR assay using additional primers derived from the partial *rep* sequence. Polymerase chain reactions were performed using the Platinum™ Taq DNA Polymerase (Invitrogen™, Thermo Fisher Scientific Corporation, Waltham, MA, USA) according to the manufacturers’ instructions. Sterile water was used as the negative control in all PCR reactions.

### 2.3. Nucleotide Sequencing

The PCR amplicons were purified using the Wizard^®^ SV Gel and PCR Clean-Up kit (Promega, Madison, WI, USA) following the instructions made available by the manufacturer. Nucleotide (nt) sequences were determined using the ABI Prism Big Dye Terminator Cycle Sequencing Ready Reaction Kit on an ABI 3730XL Genetic Analyzer (Applied Biosystems, Foster City, CA, USA).

### 2.4. Sequence Analysis

The standard BLASTN and BLASTP program (Basic Local Alignment Search Tool, www.ncbi.nlm.nih.gov/blast, accessed on 1 June 2022) was employed to conduct homology searches for related nt and deduced amino acid (aa) sequences, respectively. Putative ORFs were determined using the ORF finder (https://www.ncbi.nlm.nih.gov/orffinder/, accessed on 1 June 2022). The map of the circular viral genome of CRESS DNA virus CRESSV2/ENG22 was constructed with the ‘Draw Custom Plasmid Map’ program (https://www.rf-cloning.org/savvy.php, accessed on 6 June 2022). The putative stem-loop structure was identified in the CRESSV2/ENG22 sequence using the mFold program [41]. Pairwise sequence identities were calculated using the ‘align two or more sequences’ option of BLASTN/BLASTP (https://blast.ncbi.nlm.nih.gov/, accessed on 5 June 2022), or the EMBOSS Needle program (https://www.ebi.ac.uk/Tools/psa/emboss_needle/, accessed on 5 June 2022).

Multiple alignments of nt and deduced aa sequences were performed using the CLUSTALW (https://www.genome.jp/tools-bin/clustalw, accessed on 5 June 2022) and Clustal Omega (https://www.ebi.ac.uk/Tools/msa/clustalo/, accessed on 5 June 2022) program, respectively. The complete genomes of the PCV2 strains were evaluated for recombination events using the RDP4 program with default parameters, and were considered as recombinants if supported by two, or >two detection methods (3Seq, BOOTSCAN, CHIMAERA, GENECONV, MAXCHI, RDP, and SISCAN) with a highest acceptable *p*-value of *p* < 0.01 with Bonferroni’s correction, as described previously [38,39,42]. Phylogenetic analyses of PCV2 were carried out by both maximum-likelihood (ML) and neighbor-joining (NJ) methods using the MEGA7 software, with the Kimura 2-parameter model of substitution and 1000 bootstrap replicates, whereas phylogenetic analysis of CRESSV2/ENG22 was performed by the ML method, supported with 1000 bootstrap replicates and the LG + F + G + I model of substitution, as described in previous studies [8,15,38].

### 2.5. GenBank Accession Numbers

The GenBank accession numbers for the PCV1, PCV2, pigeon circovirus-like virus and porcine-associated CRESS DNA virus CRESSV2/ENG22 sequences determined in the study are ON813247, ON729964-ON729980, ON813248, and ON813249, respectively.

## 3. Results and Discussion

The Caribbean nation of the Dominican Republic (~18,619 square miles, human population of ~10,622,000 in the year 2022) is located on the Greater Antillean Island of Hispaniola, with Haiti to the west (Supplementary Material S1) (https://www.britannica.com/place/Dominican-Republic, accessed 11 June 2022). Pork production (~100,829,500 kgs in 2021, approximately 1,800,000 pigs reared in commercial farms and backyard systems) contributes significantly to the livestock economy and is an important source of animal protein in the Dominican Republic (https://agricultura.gob.do, accessed on 11 June 2022).

### 3.1. Detection and Complete Genome Analysis of PCV2 in Diarrheic Pigs from the Dominican Republic

In the present study, we observed high rates (47.5%, 48/101) of detection of PCV2 in diarrheic pigs from three pig farms (Cabrera in Central-North, and Pedro Brand and Villa Mella in Central-South) in the Dominican Republic (Supplementary Materials S1 and S3). The only other published study on PCV2 from the Dominican Republic reported high (59.6%, 65/109) seropositivity (antibodies to PCV2) on pig farms near the western border of the country [33]. Taken together, these observations indicated that PCV2 might be widely distributed in porcine populations in the Dominican Republic. Although the sample size varied between the three pig farms, we observed significant differences in PCV2 detection rates between the farm in Villa Mella (20%, 10/50) and those in Cabrera (80%, 28/35) and Pedro Brand (62.5%, 10/16), which might be attributed to the routine vaccination (CIRCUMVENT^®^ PCV M vaccine, Merck & Co., Inc., Rahway, NJ, USA) of pigs at Villa Mella. The high PCV2 detection rates in 2020 (74.5%, 38/51) and relatively low detection rates in 2021 (20%, 10/50) might be due to sampling on unvaccinated and vaccinated farms during 2020 and 2021, respectively. The rates of PCV2 detection in different age groups of the diarrheic pigs was 23.52% (4/17), 45.71% (16/35), 94.44% (17/18), 0% (0/1), 46.15% (6/13), 21.42% (3/14), and 66.66% (2/3) in piglets, weaners, growers, gilt, farrow/pregnant sows, dry sows, and boars, respectively (Supplementary Material S3). Three of the PCV2 positive pigs died (one grower in Cabrera, and one weaner and one dry sow in Villa Mella), and most of the PCV2 positive weaners and growers were of low body weight and showed stunted growth. Although we could not obtain specific information on the reproductive health of the PCV2 positive sows, especially pregnant animals, abortions, decreased litter size and continuous repetition of estrous were important concerns for the farms. The fecal samples were also screened for AdV and RVA as a part of another ongoing research project (detection and molecular characterization of Adv and RVA in porcine enteritis) on the farms. Adenoviral DNA was detected in 21 (43.75%) of the 48 PCV2 positive pigs, whilst none of the animals tested positive for RVAs (Supplementary Material S3).

**Figure 1:**
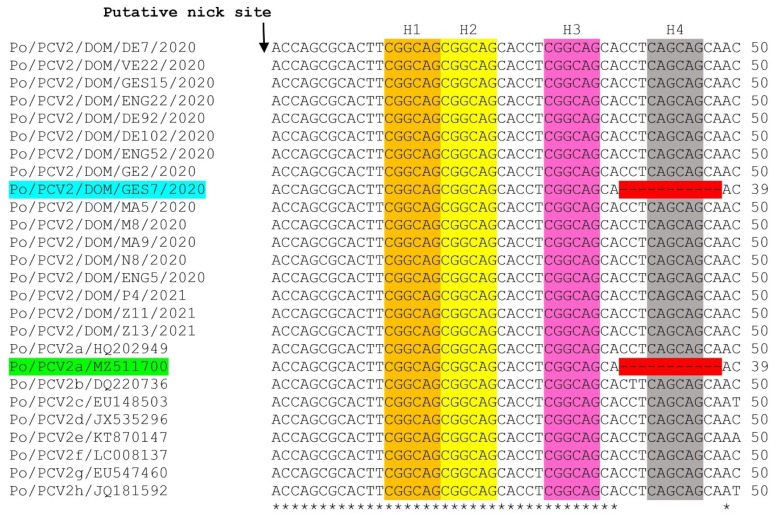
Multiple alignment of the partial origin of replication (*ori*) sequences (downstream of the putative nick site) of porcine circovirus 2 (PCV2) strains from the Dominican Republic (host/virus species/country/virus name/year) with those of reference PCV2 strains (host/PCV2 genotype/GenBank accession number). The four hexamer repeats, H1-4, are highlighted with orange, yellow, pink, and gray, respectively. A unique 11-nucleotide (nt) deletion (shown with red) was observed in the putative *ori* sequences of PCV2 strain GES7 (shown with blue) from the Dominican Republic and PCV2a strain Belgorod RA18 (GenBank accession number MZ511700, shown with green) from Russia. A ‘*’ denotes an identical nt residue, whilst ‘-’ indicates absence of an nt residue. Numbers to the right indicate the positions of the nt for respective sequences.

Based on available volumes of fecal samples, 17 PCV2 strains, representing the three pig farms, different age groups, and sampling periods, were selected for molecular characterization of the complete genome. Except for a single strain (strain Po/PCV2/DOM/ GES7/2020, 1756 bp in size), the complete genomes of PCV2 strains from the Dominican Republic were 1767 nt in length, as observed in those of most other PCV2 [1,5,11,15]. A unique 11 nt deletion was observed downstream of the putative nick site in the origin of replication (*ori*) of PCV2 strain GES7 (Figure 1). As a result, the putative *ori* of GES7 lacked the fourth hexamer (H4) repeat sequence (Figure 1). Among PCV2, this deletion appears to have only been reported in a single PCV2a strain (strain Belgorod RA18) from Russia [43]. Since the PCV Rep binds preferentially to the H1/H2 tandem, whilst H3/H4 are considered as optional binding sites, it has been proposed that H4 might not be essential for the replication of PCV [44]. This observation was corroborated by the detection of GES7 and Belgorod RA18 [43] in live pigs, although the replication of either PCV2 strain remains to be studied in vivo or in vitro.

**Figure 2:**
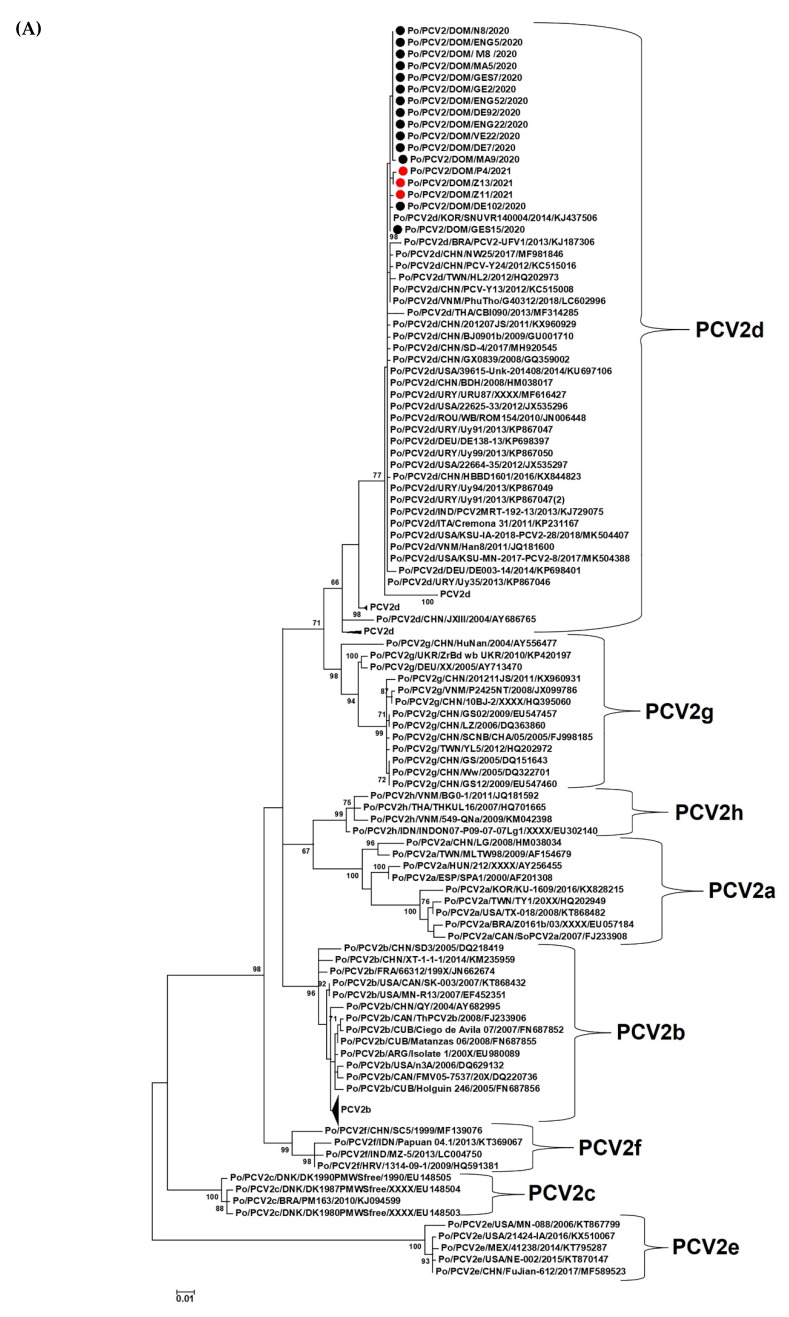

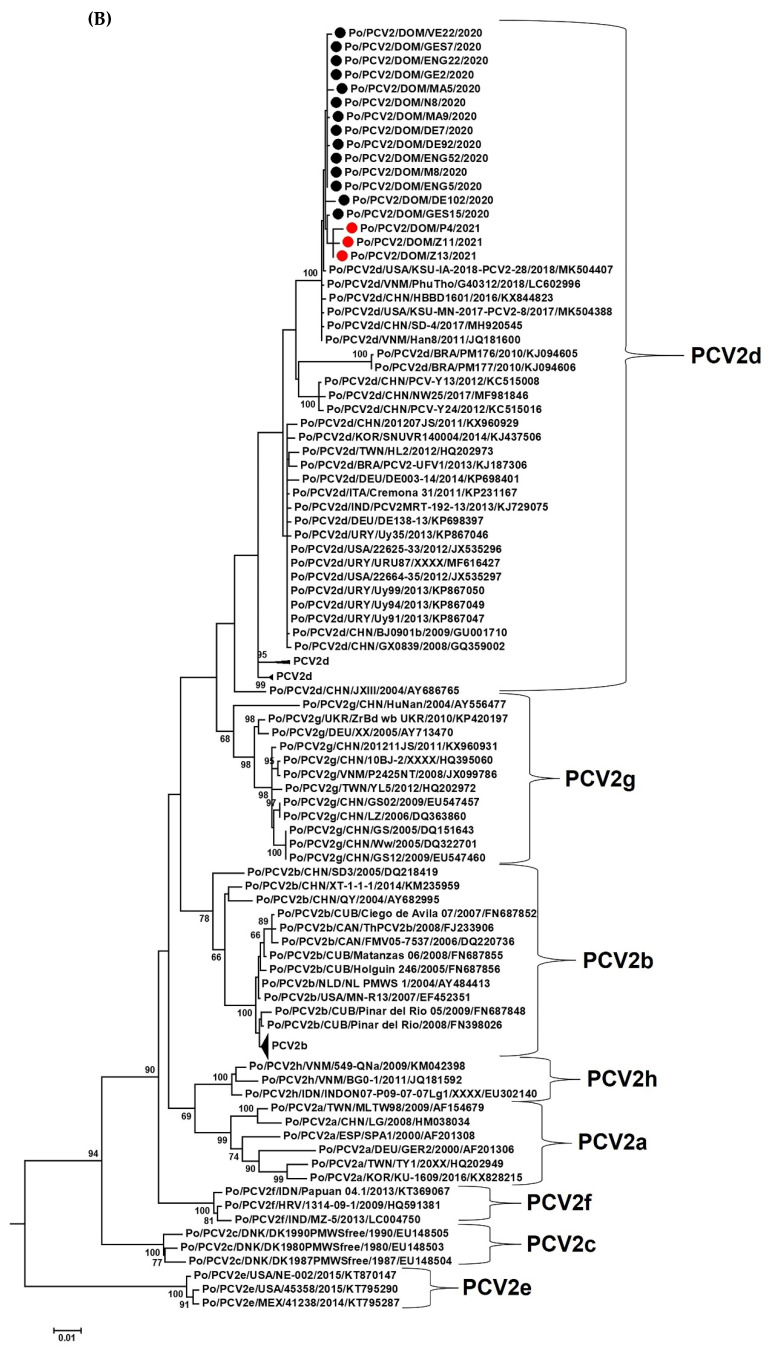
Phylogenetic analyses of the nucleotide sequences of open reading frame 2 (**A**) and complete genomes (**B**) of porcine circovirus 2 (PCV2) strains from the Dominican Republic with those of viruses belonging to the eight PCV2 genotypes (PCV2a-PCV2h). The trees were constructed using the maximum-likelihood method. The host/virus species/country/virus name/year are shown for the PCV2 strains from the Dominican Republic, whilst the host/PCV2 genotype/country/virus name/year of detection, or year of GenBank submission/GenBank accession number have been mentioned for the other PCV2 strains. Red and black circles indicate the PCV2 strains detected in vaccinated animals during 2021 and in unvaccinated animals during 2020, respectively, from the Dominican Republic. In (**B**), porcine circovirus 1 strain Po/PCV1/UK/PCV1-Eng-1970/1970/KJ408798 was used as the outgroup sequence (not shown here due to space constraints). Bootstrap values < 65% are not shown. Scale bar, 0.01 substitutions per nucleotide. Phylogenetic analyses performed using the neighbor-joining method are shown in Supplementary Material S4.

To determine the genotype nature and evolution of PCV2 in the Dominican Republic (DOM), phylogenetic analysis was performed on the ORF2 (nt 1734-nt 1030) and complete genome sequences of the 17 PCV2 strains from the present study. Since the PCV2 genotyping scheme is based on the NJ method [15], whilst the ML method is more robust than the NJ method, the DOM PCV2 sequences were phylogenetically evaluated by both methods. The ORF2 and complete genome sequences of the DOM PCV2 strains exhibited similar clustering patterns with the ML and NJ methods, forming a single cluster within the PCV2d genotype (Figure 2; Supplementary Material S4). The 17 DOM PCV2 strains shared ORF2 and complete genome nt sequence identities of 99.57–100% and 98.98–100%, respectively, between themselves. With other PCVs, the DOM PCV2 strains shared maximum ORF2 nt sequence identities of 99.86–100% with PCV2d strain 15-63-GB (GenBank accession number KX808476) from South Korea or RQ2 (KX247818) from China and maximum complete genome nt sequence identities of 99.21–99.89% with PCV2d strain PCV2/PhuTho/G40312/2018 (LC602996) from Vietnam or KSU-IA-2018-PCV2-28 (MK504407) from the USA. Considering the implications of recombination events on PCV2 evolution, especially on viral antigenicity and virulence [5,15,45], the complete genome sequences of the DOM PCV2 strains were subjected to recombination analysis. However, the RDP4 program did not provide evidence for recombination events involving the DOM PCV2 sequences (data not shown).

The putative Cap of the DOM PCV2 strains shared deduced aa identities of 99.15–100% between themselves, and maximum identities of 99.57–100% with those of other PCV2 (PCV2d) strains. The PCV2d genotype nature of the DOM strains was further corroborated by multiple alignment of the DOM Cap sequences with those of viruses representing the eight PCV2 genotypes (Supplementary Material S5). The putative Cap of the DOM PCV2 strains contained an extra aa (K) at the end (aa position 234) of the carboxyl terminus (proposed to facilitate viral attachment to host cells) and retained the signature motif (86SNPLTV91) that distinguishes PCV2d from PCV2a and PCV2b (Supplementary Material S5) [13,38,46]. Only two aa mismatches (G/R and T/A at positions 169 and 170, respectively) were observed between the Cap sequences of the DOM PCV2 strains (Supplementary Material S5). Whilst 169G, or 169R has been reported in several other PCV2d strains, the T170A substitution (observed in DOM PCV2 strain DE102) appears to have only been reported in a PCV2d strain (GenBank accession number MH323413) from China (Supplementary Material S5). Although the implications of these mutations are not known, the PCV2 Cap region spanning aa positions 169–180 has been proposed to serve as a decoy epitope, with aa residues at 170–172, 174 and 175 participating in the recognition by antibodies [47]. The putative Rep of the DOM PCV2 strains shared deduced aa identities of 98.41–100% between themselves, and maximum identities of 99.04–100% with those of other PCV2 strains. A total of 10 aa mismatches were observed between the DOM Rep sequences, of which 7 substitutions appear to be unique/rarely reported in PCV2 Rep sequences (Supplementary Material S6).

Taken together, the present study is the first report on detection and molecular characterization of PCV2 in the Dominican Republic. For the first time, PCV2d was identified as the predominant PCV2 genotype in a study from the Caribbean region. In the Americas, PCV2d has been identified as the major PCV2 genotype in the USA [48], and recently, some South American countries have reported the emergence/genotype shift to PCV2d [30,31,49,50]. On the other hand, previous studies from the Caribbean region (Cuba and St. Kitts) reported PCV2b as the sole genotype, except for identification of 3 PCV2b-PCV2d recombinant strains from St. Kitts [36,37,38]. Since the studies from Cuba and St. Kitts were based on porcine samples collected before 2010 and 2016, respectively, they might not reflect the present status of PCV2 genotypes circulating in the region [36,37,38]. The present study, based on samples collected during 2020–2021, indicated that a potential PCV2 genotype shift from PCV2b to PCV2d might be happening in the Caribbean region, which mirrored the current PCV2 genotype scenario in many other parts of the world [2,5,15]. However, large-scale molecular epidemiological studies involving several other Caribbean islands are required to confirm this observation. The emergence of PCV2d as the major PCV2 genotype, even in vaccinated farms, has sparked a debate between vaccine efficacy versus vaccination failure, especially with regards to the current PCV2a-based vaccines [5,14,18,19,20,23,24,25,28,29,30,31]. In the present study, we observed significant differences in PCV2 detection rates between the vaccinated (20%) and unvaccinated (62.5% and 80%) farms, corroborating previous observations that PCV2a-based vaccines confer protection against other PCV2 genotypes [5,14]. However, the PCV2 detection rates among diarrheic pigs was still high in the vaccinated farm, which might be related to varying levels of vaccine efficacy against heterologous genotypes, and/or other factors, such as co-infections with other pathogens, individual host factors, and husbandry practices [5]. 

Although the present study has implications on the regional pork industry and adds important information to our current knowledge on geographical distribution of PCV2 genotypes, there were a few limitations: (i) The samples were screened for PCV2 by a nested PCR, whilst qPCR has been the preferred screening assay in many other studies [17]. However, the PCV2-specific nested PCR assay employed in our study reported high PCV2 detection rates (47.5%, 48/101) in the diarrheic pigs. Nested PCR assays exhibit higher sensitivity and specificity than conventional PCRs [51], and identical PCV2 detection rates have been reported with nested PCR and qPCR assays [52]; (ii) Since the study was limited to diarrheic fecal samples, our findings may not reveal the overall PCV2 scenario in the farms; (iii) Due to lack of sufficient volumes of fecal samples, we could not sequence all the PCV2 positive samples. However, the 17 genotyped samples represented the three pig farms, different age groups of animals and sampling periods; and (iv) Since the PCV2 positive samples were screened for only two (AdV and RVA) of the several other enteric pathogens, we could not establish whether PCV2 caused diarrhea in the animals.

### 3.2. Identification of a Pigeon Circovirus-like Sequence and Complete Genome Analysis of an Unclassified CRESS DNA Virus from Diarrheic Pigs

In the present study, porcine fecal samples from Cabrera and Pedro Brand were also screened for novel circoviruses/other CRESS DNA viruses using a circovirus/cyclovirus pan-*rep* nested PCR assay. Seven samples (six of which had tested positive with the PCV2-specific nested-PCR (Supplementary Material S3)) yielded the expected ~400 bp amplicon and were sequenced for the partial *rep* gene. By BLASTN analysis of the partial *rep* sequences, ENG10, GE2, GES4, and GES72 shared maximum homology with PCV2 strains, whilst VE22 was closely related to PCV1 strains. ENG7 shared maximum nt sequence identity of 92.49% with circovirus strains (GenBank accession numbers JN183455, MW181954, and MW181966-67) from pigeons in China. A previous study speculated that PCV2 was transmitted to wild boars from avian species, and subsequently infected domestic pigs [53]. Therefore, it would have been interesting to study the evolution of the pigeon circovirus-like viral strain from a diarrheic pig. However, despite repeated efforts, we could not amplify the complete genome of the pigeon circovirus-like virus in sample ENG7. Since the pigeon circovirus-like sequence was detected in a fecal sample, we could not determine whether the virus infected pigs, or was of dietary origin (consumption of avian feces). The partial *rep* sequence in ENG22 shared maximum homology with unclassified CRESS DNA viral sequences from porcine feces [8] and was eventually characterized for the complete genome.

**Figure 3:**
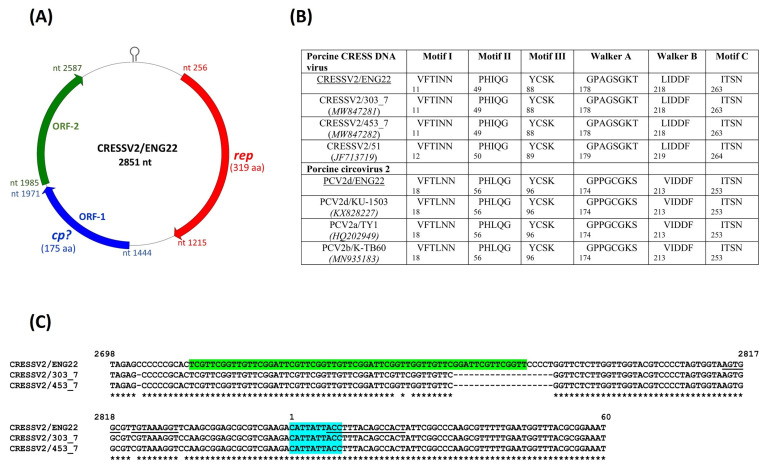
Genome organization of porcine-associated circular Rep-encoding single-stranded (CRESS) DNA virus CRESSV2/ENG22. The nonanucleotide motif at the apex of a putative stem-loop structure (marked in the large intergenic region) and the three predicted open reading frames (ORFs) were located on the genome-sense strand of CRESSV2/ENG22. The ORF encoding the putative replication associated (Rep) protein is shown with red, whilst ORF1 (speculated to encode the putative capsid (Cp) protein) and ORF2 is shown with blue and green, respectively (**A**). Presence of the conserved rolling circle replication (motifs I through III) and superfamily 3 helicase (Walker A and B, and motif C) motifs in the putative Rep proteins of CRESSV2/ENG22, other porcine-associated CRESS DNA viruses, and porcine circovirus 2. The number below the motif denotes the position of the amino acid (aa) residue in the respective Rep protein. Sample ENG22 also tested positive for porcine circovirus 2d. The GenBank accession numbers are shown in parentheses (**B**). The putative nonanucleotide motif (highlighted with blue), complementary sequences flanking the nonanucleotide motif (underlined), and repeat sequences (shown with green) in the large intergenic region of porcine-associated CRESS DNA viruses CRESSV2/ENG22, CRESSV2/303_7 (GenBank accession number MW847281), and CRESSV2/453_7 (MW847282). A ‘*’ denotes an identical aa residue, whilst ‘-’ indicates absence of an aa residue. The numbers correspond to the positions of nucleotides in the complete genome sequence of CRESSV2/ENG22 (**C**).

**Figure 4:**
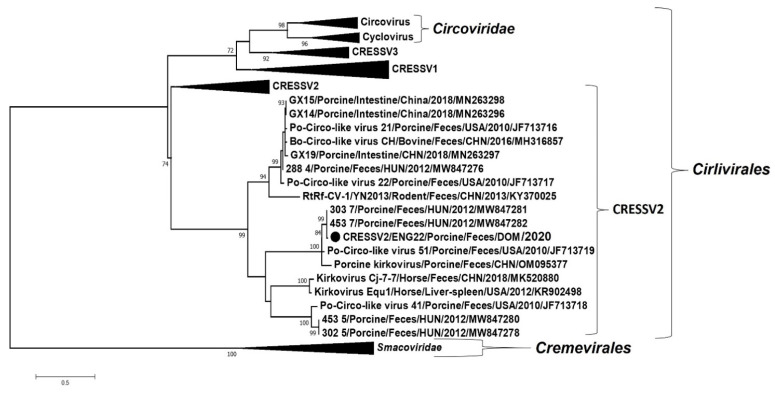
Phylogenetic analysis of the putative replication associated (Rep) protein of porcine-associated circular Rep-encoding single-stranded (CRESS) DNA virus CRESSV2/ENG22 (shown with black circle) with those of viruses belonging to the orders *Cremevirales* and *Cirlivirales*. The virus name/source: animal/sample/country/year/GenBank accession number are shown for the CRESSV2 DNA viruses. Scale bar, 0.5 substitutions per amino acid residue. Bootstrap values of <70 are not shown.

The complete genome of the CRESS DNA virus (CRESSV2/ENG22) in sample ENG22 was 2851 nt in length, and shared maximum nt sequence identities of ~93% with those of porcine-associated CRESS DNA viruses 303_7 and 453_7 from porcine feces in Hungary [8]. Based on the genome organization, CRESSV2/ENG22 was assigned to type V CRESS DNA genomes (Figure 3) [54]. At least three putative ORFs (*rep*, ORF1 and ORF2) were located on the genome-sense strand of CRESSV2/ENG22 (Figure 3). The *ori* (located in the large intergenic region (LIR)) of CRESS DNA viruses is characterized by a conserved nonanucleotide motif at the apex of a stem loop structure, and presence of repeat elements [8,55]. The LIR (between rep and ORF2) of CRESSV2/ENG22 consisted of a putative nonanucleotide motif (CATTATTACC) that was surrounded with 15 nt-long complementary sequences, indicating potential loop formation (Figure 3). A 64 nt-long region of repeat elements was observed upstream of the nonanucleotide motif (Figure 3). Interestingly, the LIR of CRESSV2/ENG22 contained a 19 nt-long region (that included repeat elements) upstream of the nonanucleotide motif which was lacking in the related porcine-associated CRESS DNA viral sequences (Figure 3). The putative Rep of CRESSV2/ENG22 retained the rolling circle replication and superfamily 3 helicase motifs that are conserved in other CRESS DNA viruses (Figure 3; Supplementary Material S7) [55], and shared maximum deduced aa identities of 98.75% with the Rep of 303_7 and 453_7. Phylogenetically, CRESSV2/ENG22 grouped with 303_7 and 453_7 within a large cluster that predominantly consisted of several diverse porcine-associated unclassified CRESSV2 genomes belonging to the order *Cirlivirales* (Figure 4). The amino terminus of the putative ORF1 product of CRESSV2/ENG22 accumulated slightly more arginine residues, corroborating previous speculations that ORF1 might encode the capsid protein (Supplementary Material S8) [8,55]. The putative ORF1 product of CRESSV2/ENG22 shared maximum deduced aa identities of 99.43% with those of 303_7 and 453_7.

Although the circovirus/cyclovirus pan-*rep* nested PCR assay employed in the present study has been successfully used to identify novel CRESS DNA viruses [39,40,55,56], we detected unclassified CRESS DNA viruses in a single porcine fecal sample. This might suggest that porcine-associated CRESS DNA viruses are circulating at low frequencies in the sampled farms. On the other hand, a recent study from Hungary, based on metagenomic analysis, reported high detection rates (86.9%, 20/23 samples) of porcine circovirus-like CRESS DNA viral sequences in stool samples [8], indicating that next generation sequencing technologies might allow a broader-spectrum detection of porcine-associated novel CRESS DNA viruses compared to the circovirus/cyclovirus pan-*rep* nested PCR assays. The complete genomic analysis of CRESSV2/ENG22 suggested that related unclassified CRESS DNA viruses are circulating among pigs in different geographical regions, indicating possible spread of the virus, or parallel evolutionary events [8]. Although the porcine-associated CRESS DNA viruses have been proposed to be derived from the gut microbiome/diet/environmental contamination, their detection in diseased animals warrants further investigation [8,9,10,54,55].

## 4. Conclusions

The present study is the first to report high rates of detection (47.5%, 48/101) and molecular characterization of PCV2 in diarrheic pigs from the Dominican Republic, warranting large-scale molecular epidemiological studies on PCV2 in pig farms and backyard systems across the country. Based on analysis of viral genomes of the 17 PCV2 positive samples that were representative of sampling sites, age groups of animals, and sampling periods, PCV2d was identified as the predominant/sole genotype. To our knowledge, this is the first report on predominance of PCV2d from a Caribbean country, suggesting that a genotype shift from PCV2b to PCV2d might be happening in the Caribbean region, which reflected the current PCV2 genotype scenario in many other parts of the world [2,5,14,15,16,17]. Significant differences were observed in PCV2 detection rates between the vaccinated farm and unvaccinated farms, emphasizing the importance of vaccination against PCV2 (including heterologous strains) in porcine populations. The identification of a pigeon circovirus-like virus and an unclassified CRESS DNA virus highlighted the diversity of the CRESS DNA viruses in porcine fecal samples, warranting further studies on the epidemiology and biology of these novel viruses.

## Supplementary Materials

The following supporting information can be downloaded at: https://www.mdpi.com/article/10.3390/v14081799/s1, Supplementary Material S1: Map of the Dominican Republic showing the locations of the three pig farms that were sampled in the study; Supplementary Material S2: Six primer pairs were used in three overlapping nested PCR assays to amplify the complete genomes of porcine circovirus 2 (PCV2) strains from the Dominican Republic. Nested PCR reaction ‘A’ was used to screen the porcine fecal samples for PCV2; Supplementary Material S3: Details of the diarrheic pigs that tested positive for porcine circovirus 2 (PCV2) in the Dominican Republic; Supplementary Material S4: Phylogenetic analyses of the nucleotide sequences of open reading frame 2 (A) and complete genomes (B) of porcine circovirus 2 (PCV2) strains from the Dominican Republic with those of viruses belonging to the eight PCV2 genotypes (PCV2a-PCV2h); Supplementary Material S5: Multiple alignment of the putative capsid proteins of porcine circovirus 2 (PCV2) strains from the Dominican Republic with those of virus strains representing the 8 PCV2 genotypes (PCV2a-h); Supplementary Material S6: Multiple alignment of the putative replication-associated proteins (Rep) of porcine circovirus 2 (PCV2) strains from the Dominican Republic with those of virus strains representing the 8 PCV2 genotypes (PCV2a-h); Supplementary Material S7: Multiple alignment of the putative replication-associated proteins (Rep) of porcine-associated CRESS DNA virus CRESSV2/ENG22, other porcine-associated CRESS DNA viruses (CRESS group/virus name/GenBank accession number), and porcine circovirus 2 strains (PCV2 genotype/virus name/GenBank accession number); Supplementary Material S8: Multiple alignment of the putative protein encoded by open reading frame 1 (ORF1) of porcine-associated CRESS DNA virus CRESSV2/ENG22 and other porcine-associated CRESS DNA viruses (virus name/GenBank accession number).
