# Supplementary material for: Detection and Complete Genome Analysis of Porcine Circovirus 2 (PCV2) and an Unclassified CRESS DNA Virus from Diarrheic Pigs in the Dominican Republic: First Evidence for Predominance of PCV2d from the Caribbean Region
Source: Viruses. 2022 Aug 17;14(8):1799. doi: 10.3390/v14081799 (PMC9415081; doi:10.3390/v14081799)
Supplement: Supplementary file 1 [file viruses-14-01799-s001.zip › Supplementary material S1.pdf]

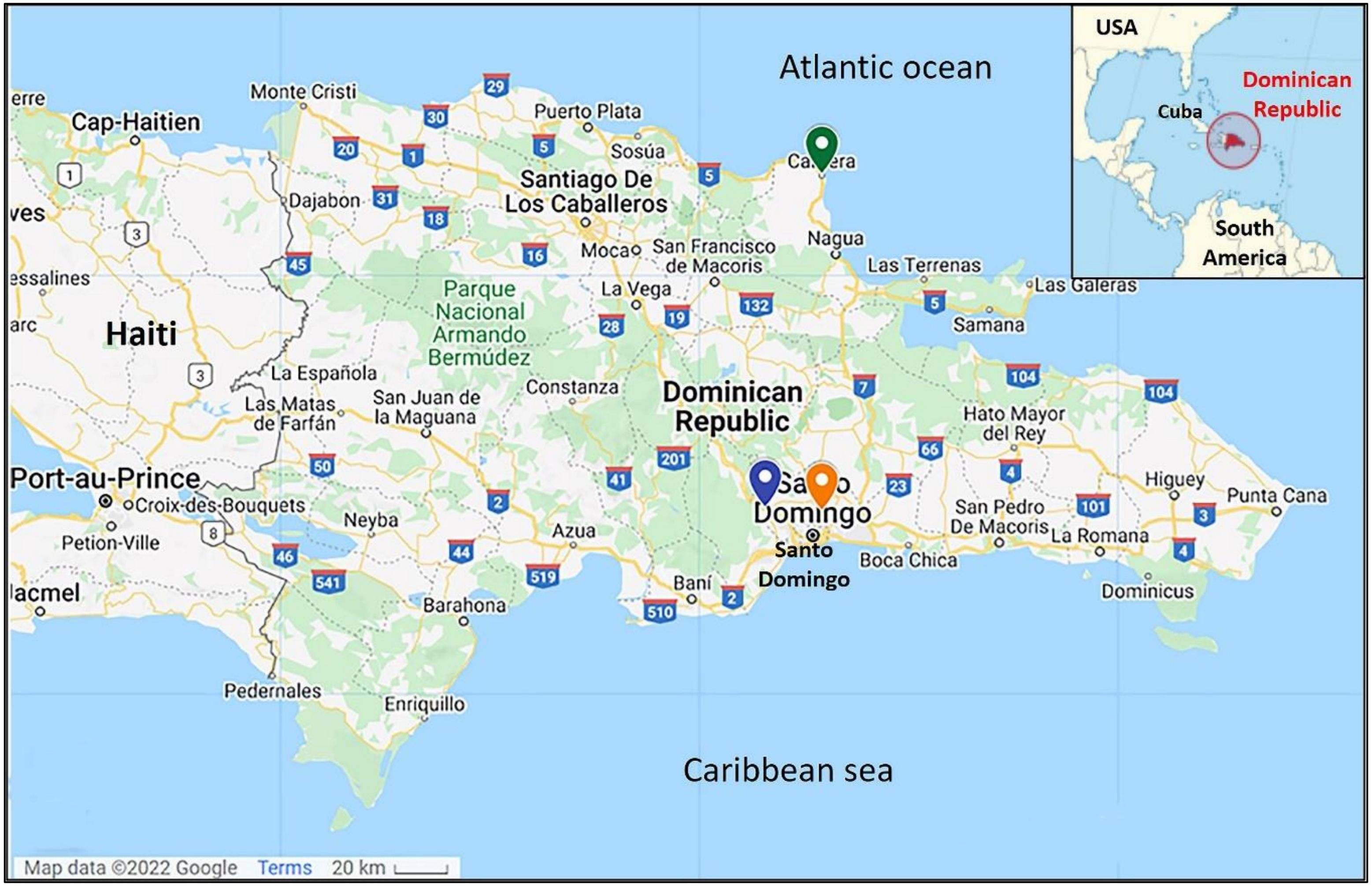

Supplementary material S1. Map of the Dominican Republic showing the locations of the three pig farms that were sampled in the study. The pig farm in the municipality of Cabrera, Pedro Brand, and Villa Mella is shown with green, blue, and orange pins, respectively. The map was adapted from https://www.google.com/maps (accessed June 2, 2022). ***Inset:*** Geographical location of the Dominican Republic (encircled and shown with red) in the Caribbean region. The map was adapted from https://commons.wikimedia.org/wiki/File:Dominican_Republic_in_the_world_(W3).svg (author: TUBS, https://commons.wikimedia.org/wiki/User:TUBS) on June 2, 2022, and is licensed for free sharing and adaptation under the Creative Commons Attribution-Share Alike 3.0 Unported license.
