# Supplementary material for: Detection and Complete Genome Analysis of Porcine Circovirus 2 (PCV2) and an Unclassified CRESS DNA Virus from Diarrheic Pigs in the Dominican Republic: First Evidence for Predominance of PCV2d from the Caribbean Region
Source: Viruses. 2022 Aug 17;14(8):1799. doi: 10.3390/v14081799 (PMC9415081; doi:10.3390/v14081799)
Supplement: Supplementary file 1 [file viruses-14-01799-s001.zip › Supplementary material S2.pdf]

Supplementary material S2. Six primer pairs were used in three overlapping nested PCR assays to amplify the complete genomes of porcine circovirus 2 (PCV2) strains from the Dominican Republic. Nested PCR reaction 'A' was used to screen the porcine fecal samples for PCV2.

**Nested PCR reaction**		**Primer name**	**Primer sequence** [1] **(5′-3′)**	**Primer position** [2]
**A**	First PCR	PCV2-F-A<br>PCV2-R-A	GACCCCAACCCCATAAAAGGTGG<br>CCATTCCAACGGGGTCTGATTGC	nt 79-nt 101<br>nt 836-nt 814
	Second PCR	PCV2-FN-A<br>PCV2-RN-A	GAAGACGAGCGCAAGAAAATACG<br>GTTCCACCTTTAGTCTCTACAGTC	nt 126-nt 148<br>nt 778-nt 755
**B**	First PCR	PCV2-F-B<br>PCV2-R-B	GGCTGCTAATTTTGCAGACCCGG<br>TCCCCGATCACCCAGGGTGAC	nt 559-nt 621<br>nt 1410-nt 1390
	Second PCR	PCV2-FN-B<br>PCV2-RN-B	GGAAACCACCTAGAAACAAGTG<br>GCTCCAGTGCTGTTATTCTAG	nt 634-nt 655<br>nt 1379-nt 1359
**C**	First PCR	PCV2-F-C<br>PCV2-R-C	GTTTGTAGTCTCAGCCACAGCTG<br>CGGGCACCCAAATACCACTTCAC	nt 1169-nt 1191<br>nt 289-nt 267
	Second PCR	PCV2-FN-C<br>PCV2-RN-C	GTGGAATCTAGGACAGGTTTGG<br>CTGGAGGTGAGGTGTTCGTC	nt 1226-nt 1247<br>nt 227-nt 208

[1] The primers were derived from conserved regions among PCV2 sequences representing the different PCV2 genotypes.

[2] Nucleotide (nt) positions are those of reference PCV2b strain Po/PCV2b/CAN/FMV05-7537/20XX, GenBank accession number DQ220736.
