# Supplementary material for: Detection and Complete Genome Analysis of Porcine Circovirus 2 (PCV2) and an Unclassified CRESS DNA Virus from Diarrheic Pigs in the Dominican Republic: First Evidence for Predominance of PCV2d from the Caribbean Region
Source: Viruses. 2022 Aug 17;14(8):1799. doi: 10.3390/v14081799 (PMC9415081; doi:10.3390/v14081799)
Supplement: Supplementary file 1 [file viruses-14-01799-s001.zip › Supplementary material S3.pdf]

Supplementary material S3. Details of the diarrheic pigs that tested positive for porcine circovirus 2 (PCV2) in the Dominican Republic.

Animal/sample number	Age group/category of animal [1]	Location of the farm	Year of sample collection	Vaccination status against PCV2 [2]	Coinfection with	
					Adenovirus [3]	*Rotavirus A* [4]
DE3	Weaner	Cabrera [5]	2020	Not vaccinated	Negative	Negative
DE7	Weaner	Cabrera	2020	Not vaccinated	Negative	Negative
DE8	Weaner	Cabrera	2020	Not vaccinated	Negative	Negative
DE10	Weaner	Pedro Brand [5]	2020	Not vaccinated	Positive	Negative
DE11	Weaner	Pedro Brand	2020	Not vaccinated	Negative	Negative
DE22	Weaner	Cabrera	2020	Not vaccinated	Positive	Negative
DE32	Weaner	Cabrera	2020	Not vaccinated	Negative	Negative
DE83	Weaner	Cabrera	2020	Not vaccinated	Negative	Negative
DE92	Weaner	Pedro Brand	2020	Not vaccinated	Positive	Negative
DE102	Weaner	Pedro Brand	2020	Not vaccinated	Negative	Negative
DES1	Weaner	Cabrera	2020	Not vaccinated	Negative	Negative
DES5	Weaner	Cabrera	2020	Not vaccinated	Negative	Negative
DES9	Weaner	Pedro Brand	2020	Not vaccinated	Negative	Negative
ENG2	Grower	Cabrera	2020	Not vaccinated	Positive	Negative
ENG3	Grower	Cabrera	2020	Not vaccinated	Negative	Negative
ENG4	Grower	Cabrera	2020	Not vaccinated	Positive	Negative
ENG5	Grower	Cabrera	2020	Not vaccinated	Positive	Negative
ENG6	Grower	Cabrera	2020	Not vaccinated	Negative	Negative
ENG9	Grower	Cabrera	2020	Not vaccinated	Negative	Negative
ENG10	Grower	Cabrera	2020	Not vaccinated	Negative	Negative
ENG11	Grower	Cabrera	2020	Not vaccinated	Negative	Negative
ENG12	Grower	Cabrera	2020	Not vaccinated	Negative	Negative
ENG14	Grower	Cabrera	2020	Not vaccinated	Positive	Negative
ENG19	Grower	Cabrera	2020	Not vaccinated	Negative	Negative
ENG21	Grower	Cabrera	2020	Not vaccinated	Positive	Negative
ENG22	Grower	Cabrera	2020	Not vaccinated	Negative	Negative
ENG52	Grower	Cabrera	2020	Not vaccinated	Positive	Negative
GE2	Farrow [6]/Pregnant sow	Pedro Brand	2020	Not vaccinated	Negative	Negative
GES4	Farrow/Pregnant sow	Pedro Brand	2020	Not vaccinated	Positive	Negative
GES7	Farrow/Pregnant sow	Pedro Brand	2020	Not vaccinated	Positive	Negative
GES15	Farrow/Pregnant sow	Pedro Brand	2020	Not vaccinated	Positive	Negative
GES72	Farrow/Pregnant sow	Pedro Brand	2020	Not vaccinated	Negative	Negative
M8	Piglet	Cabrera	2020	Not vaccinated	Positive	Negative
MA5	Piglet	Cabrera	2020	Not vaccinated	Positive	Negative
MA9	Piglet	Cabrera	2020	Not vaccinated	Positive	Negative
N8	Grower	Cabrera	2020	Not vaccinated	Positive	Negative
VE8	Boar	Cabrera	2020	Not vaccinated	Positive	Negative
VE22	Boar	Cabrera	2020	Not vaccinated	Positive	Negative
D6	Weaner	Villa Mella [5]	2021	Vaccinated	Negative	Negative
D17	Weaner	Villa Mella	2021	Vaccinated	Positive	Negative
D18	Weaner	Villa Mella	2021	Vaccinated	Negative	Negative
G1	Farrow/Pregnant sow	Villa Mella	2021	Vaccinated	Negative	Negative
P4	Piglet	Villa Mella	2021	Vaccinated	Negative	Negative
PP5	Dry Sow	Villa Mella	2021	Vaccinated	Negative	Negative
PP11	Dry Sow	Villa Mella	2021	Vaccinated	Negative	Negative
PP15	Dry Sow	Villa Mella	2021	Vaccinated	Negative	Negative
Z11	Grower	Villa Mella	2021	Vaccinated	Positive	Negative
Z13	Grower	Villa Mella	2021	Vaccinated	Positive	Negative

[1] Based on porcine age groups/animal categories defined by the National Farm Animal Care Council (NFACC), Canada (https://www.nfacc.ca/codes-of-practice/pig-code#glossary , accessed June 5, 2022).

[2] The animals were vaccinated with CIRCUMVENT® PCV M (Merck & Co., Inc., Rahway, NJ, USA) following the instructions of the manufacturer.

[3] Fecal samples were screened for adenoviruses using a broad-range nested PCR assay (based on the DNA-dependent DNA polymerase (*pol*) gene), as described previously [1, 2], and a porcine adenovirus-specific nested PCR assay targeting the *pol* gene (primer sequences available upon request).

[4] Screening for Rotavirus A (RVA) was performed using a RVA VP6-specific RT-PCR assay, as outlined in a previous study [3].

[5] Municipality in Dominican Republic.

[6] Refers to a sow that has recently farrowed (given birth to piglets).

References:

1. Gainor, K.; Becker, A.A.M.J.; Malik, Y.S.; Ghosh, S. First Report on Detection and Molecular Characterization of Adenoviruses in the Small Indian Mongoose (Urva Auropunctata). Viruses 2021, 13, 2194, doi:10.3390/v13112194.
2. Wellehan, J.F.X.; Johnson, A.J.; Harrach, B.; Benkö, M.; Pessier, A.P.; Johnson, C.M.; Garner, M.M.; Childress, A.; Jacobson, E.R. Detection and Analysis of Six Lizard Adenoviruses by Consensus Primer PCR Provides Further Evidence of a Reptilian Origin for the Atadenoviruses. Journal of Virology 2004, 78, 13366–13369, doi:10.1128/JVI.78.23.13366-13369.2004.
3. Navarro, R.; Aung, M.S.; Cruz, K.; Ketzis, J.; Gallagher, C.A.; Beierschmitt, A.; Malik, Y.S.; Kobayashi, N.; Ghosh, S. Whole Genome Analysis Provides Evidence for Porcine-to-Simian Interspecies Transmission of Rotavirus-A. Infection, Genetics and Evolution 2017, 49, 21–31, doi:10.1016/j.meegid.2016.12.026.
