# Supplementary material for: Detection and Complete Genome Analysis of Porcine Circovirus 2 (PCV2) and an Unclassified CRESS DNA Virus from Diarrheic Pigs in the Dominican Republic: First Evidence for Predominance of PCV2d from the Caribbean Region
Source: Viruses. 2022 Aug 17;14(8):1799. doi: 10.3390/v14081799 (PMC9415081; doi:10.3390/v14081799)
Supplement: Supplementary file 1 [file viruses-14-01799-s001.zip › Supplementary material S4.pdf]

Supplementary material S4. Phylogenetic analyses of the nucleotide sequences of open reading frame 2 **(A)** and complete genomes **(B)** of porcine circovirus 2 (PCV2) strains from the Dominican Republic with those of viruses belonging to the eight PCV2 genotypes (PCV2a-PCV2h). The trees were constructed using the neighbor-joining method, with the Kimura 2-parameter model of substitution and 1000 bootstrap replicates. The host/virus species/country/virus name/year are shown for the PCV2 strains from Dominican Republic, whilst the host/PCV2 genotype/country/virus name/year of detection, or year of GenBank submission/GenBank accession number have been mentioned for the other PCV2 strains. Red and black circles indicate the PCV2 strains detected in vaccinated animals during 2021 and in unvaccinated animals during 2020, respectively, from the Dominican Republic. In figure S4 (B), porcine circovirus 1 strain Po/PCV1/UK/PCV1-Eng-1970/1970/KJ408798 was used as the outgroup sequence (not shown here due to space constraints). Bootstrap values < 65% are not shown. Scale bar, 0.01 substitutions per nucleotide. Phylogenetic analyses performed using the maximum-likelihood method are shown in figure 2.

(A)

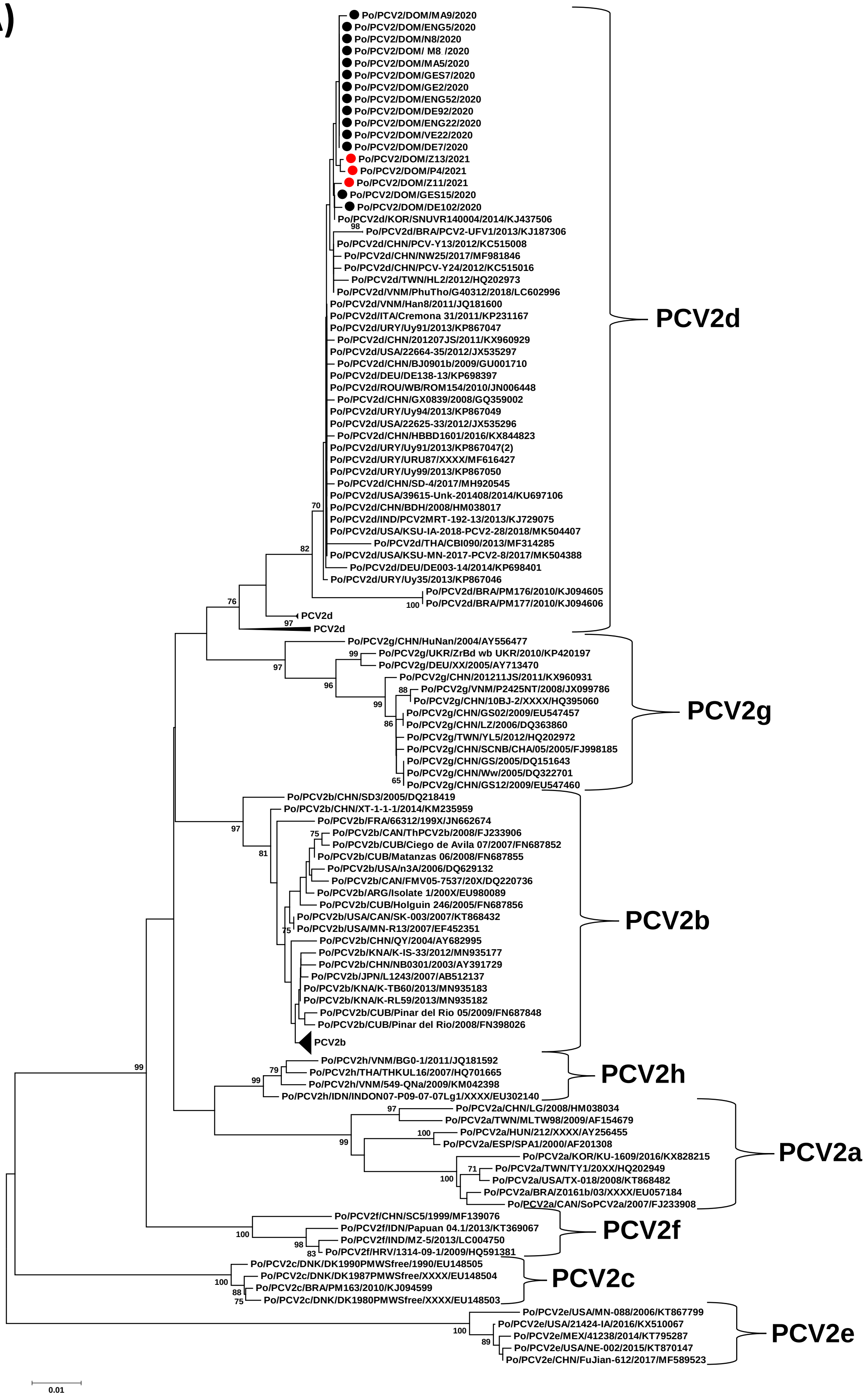

(B)

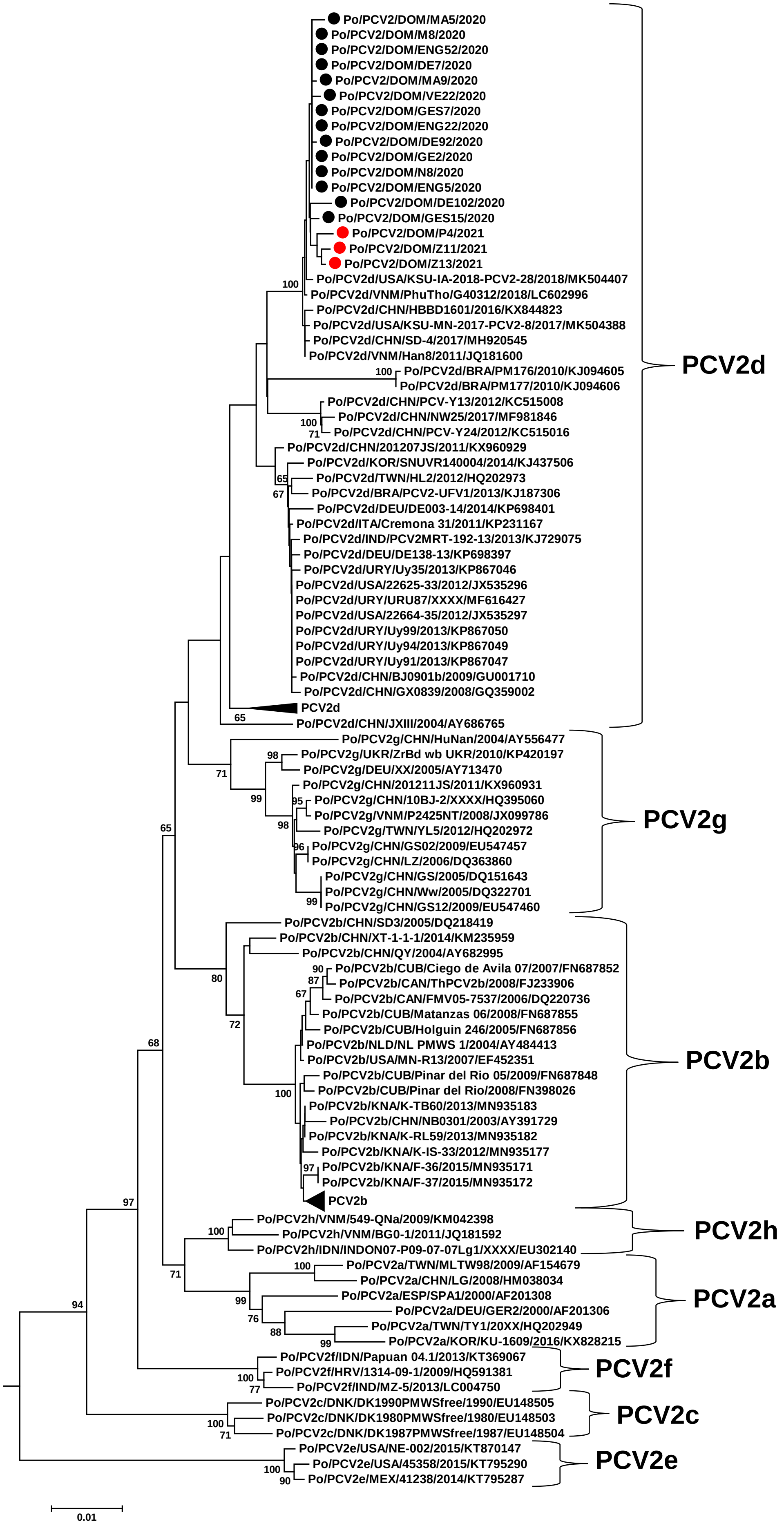
Po/PCV2/DOM/MA5/2020
Po/PCV2/DOM/M8/2020
Po/PCV2/DOM/ENG52/2020
Po/PCV2/DOM/DE7/2020
Po/PCV2/DOM/MA9/2020
Po/PCV2/DOM/VE22/2020
Po/PCV2/DOM/GES7/2020
Po/PCV2/DOM/ENG22/2020
Po/PCV2/DOM/DE92/2020
Po/PCV2/DOM/GE2/2020
Po/PCV2/DOM/N8/2020
Po/PCV2/DOM/ENG5/2020
Po/PCV2/DOM/DE102/2020
Po/PCV2/DOM/GES15/2020
Po/PCV2/DOM/P4/2021
Po/PCV2/DOM/Z11/2021
Po/PCV2/DOM/Z13/2021
Po/PCV2d/USA/KSU-IA-2018-PCV2-28/2018/MK504407
Po/PCV2d/VNM/PhuTho/G40312/2018/LC602996
Po/PCV2d/CHN/HBBD1601/2016/KX844823
Po/PCV2d/USA/KSU-MN-2017-PCV2-8/2017/MK504388
Po/PCV2d/CHN/SD-4/2017/MH920545
Po/PCV2d/VNM/Han8/2011/JQ181600
Po/PCV2d/BRA/PM176/2010/KJ094605
Po/PCV2d/BRA/PM177/2010/KJ094606
Po/PCV2d/CHN/PCV-Y13/2012/KC515008
Po/PCV2d/CHN/NW25/2017/MF981846
Po/PCV2d/CHN/PCV-Y24/2012/KC515016
Po/PCV2d/CHN/201207JS/2011/KX960929
Po/PCV2d/KOR/SNUVR140004/2014/KJ437506
Po/PCV2d/TWN/HL2/2012/HQ202973
Po/PCV2d/BRA/PCV2-UFV1/2013/KJ187306
Po/PCV2d/DEU/DE003-14/2014/KP698401
Po/PCV2d/ITA/Cremona 31/2011/KP231167
Po/PCV2d/IND/PCV2MRT-192-13/2013/KJ729075
Po/PCV2d/DEU/DE138-13/KP698397
Po/PCV2d/URY/Uy35/2013/KP867046
Po/PCV2d/USA/22625-33/2012/JX535296
Po/PCV2d/URY/URU87/XXXX/MF616427
Po/PCV2d/USA/22664-35/2012/JX535297
Po/PCV2d/URY/Uy99/2013/KP867050
Po/PCV2d/URY/Uy94/2013/KP867049
Po/PCV2d/URY/Uy91/2013/KP867047
Po/PCV2d/CHN/BJ0901b/2009/GU001710
Po/PCV2d/CHN/GX0839/2008/GQ359002
PCV2d
Po/PCV2d/CHN/JXIII/2004/AY686765
PCV2d
Po/PCV2g/CHN/HuNan/2004/AY556477
Po/PCV2g/UKR/ZrBd wb UKR/2010/KP420197
Po/PCV2g/DEU/XX/2005/AY713470
Po/PCV2g/CHN/201211JS/2011/KX960931
Po/PCV2g/CHN/10BJ-2/XXXX/HQ395060
Po/PCV2g/VNM/P2425NT/2008/JX099786
Po/PCV2g/TWN/YL5/2012/HQ202972
Po/PCV2g/CHN/GS02/2009/EU547457
Po/PCV2g/CHN/LZ/2006/DQ363860
Po/PCV2g/CHN/GS/2005/DQ151643
Po/PCV2g/CHN/Ww/2005/DQ322701
Po/PCV2g/CHN/GS12/2009/EU547460
PCV2g
Po/PCV2b/CHN/SD3/2005/DQ218419
Po/PCV2b/CHN/XT-1-1-1/2014/KM235959
Po/PCV2b/CHN/QY/2004/AY682995
Po/PCV2b/CUB/Ciego de Avila 07/2007/FN687852
Po/PCV2b/CAN/ThPCV2b/2008/FJ233906
Po/PCV2b/CAN/FMV05-7537/2006/DQ220736
Po/PCV2b/CUB/Matanzas 06/2008/FN687855
Po/PCV2b/CUB/Holguin 246/2005/FN687856
Po/PCV2b/NLD/NL PMWS 1/2004/AY484413
Po/PCV2b/USA/MN-R13/2007/EF452351
Po/PCV2b/CUB/Pinar del Rio 05/2009/FN687848
Po/PCV2b/CUB/Pinar del Rio/2008/FN398026
Po/PCV2b/KNA/K-TB60/2013/MN935183
Po/PCV2b/CHN/NB0301/2003/AY391729
Po/PCV2b/KNA/K-RL59/2013/MN935182
Po/PCV2b/KNA/K-IS-33/2012/MN935177
Po/PCV2b/KNA/F-36/2015/MN935171
Po/PCV2b/KNA/F-37/2015/MN935172
PCV2b
PCV2b
Po/PCV2h/VNM/549-QNa/2009/KM042398
Po/PCV2h/VNM/BG0-1/2011/JQ181592
Po/PCV2h/IDN/INDON07-P09-07-07Lg1/XXXX/EU302140
PCV2h
Po/PCV2a/TWN/MLTW98/2009/AF154679
Po/PCV2a/CHN/LG/2008/HM038034
Po/PCV2a/ESP/SPA1/2000/AF201308
Po/PCV2a/DEU/GER2/2000/AF201306
Po/PCV2a/TWN/TY1/20XX/HQ202949
Po/PCV2a/KOR/KU-1609/2016/KX828215
PCV2a
Po/PCV2f/IDN/Papuan 04.1/2013/KT369067
Po/PCV2f/HRV/1314-09-1/2009/HQ591381
Po/PCV2f/IND/MZ-5/2013/LC004750
PCV2f
Po/PCV2c/DNK/DK1990PMWSfree/1990/EU148505
Po/PCV2c/DNK/DK1980PMWSfree/1980/EU148503
Po/PCV2c/DNK/DK1987PMWSfree/1987/EU148504
PCV2c
Po/PCV2e/USA/NE-002/2015/KT870147
Po/PCV2e/USA/45358/2015/KT795290
Po/PCV2e/MEX/41238/2014/KT795287
PCV2e
0.01
