# Supplementary material for: Detection and Complete Genome Analysis of Porcine Circovirus 2 (PCV2) and an Unclassified CRESS DNA Virus from Diarrheic Pigs in the Dominican Republic: First Evidence for Predominance of PCV2d from the Caribbean Region
Source: Viruses. 2022 Aug 17;14(8):1799. doi: 10.3390/v14081799 (PMC9415081; doi:10.3390/v14081799)
Supplement: Supplementary file 1 [file viruses-14-01799-s001.zip › Supplementary material S5.pdf]

Supplementary material S5. Multiple alignment of the putative capsid proteins of porcine circovirus 2 (PCV2) strains from the Dominican Republic with those of virus strains representing the 8 PCV2 genotypes (PCV2a-h). Amino acid (aa) mismatches are highlighted with green. The host/virus species/country/virus name/year are shown for the PCV2 strains from the Dominican Republic, whilst the host/PCV2 genotype/GenBank accession number have been mentioned for the reference PCV2 strains. A '*' denotes an identical aa residue, whilst '-' indicates absence of an aa residue. Numbers to the right indicate the positions of the aa for respective PCV2 strains.

```
Po/PCV2/DOM/DE7/2020       MTYPRRRFRRRRHRPRSHLGQILRRRPWLVHPRHRYRWRRKNGIFNTRLSRTIGYTVKKT    60
Po/PCV2/DOM/VE22/2020      MTYPRRRFRRRRHRPRSHLGQILRRRPWLVHPRHRYRWRRKNGIFNTRLSRTIGYTVKKT    60
Po/PCV2/DOM/GES15/2020     MTYPRRRFRRRRHRPRSHLGQILRRRPWLVHPRHRYRWRRKNGIFNTRLSRTIGYTVKKT    60
Po/PCV2/DOM/ENG22/2020     MTYPRRRFRRRRHRPRSHLGQILRRRPWLVHPRHRYRWRRKNGIFNTRLSRTIGYTVKKT    60
Po/PCV2/DOM/DE92/2020      MTYPRRRFRRRRHRPRSHLGQILRRRPWLVHPRHRYRWRRKNGIFNTRLSRTIGYTVKKT    60
Po/PCV2/DOM/DE102/2020     MTYPRRRFRRRRHRPRSHLGQILRRRPWLVHPRHRYRWRRKNGIFNTRLSRTIGYTVKKT    60
Po/PCV2/DOM/ENG52/2020     MTYPRRRFRRRRHRPRSHLGQILRRRPWLVHPRHRYRWRRKNGIFNTRLSRTIGYTVKKT    60
Po/PCV2/DOM/GE2/2020       MTYPRRRFRRRRHRPRSHLGQILRRRPWLVHPRHRYRWRRKNGIFNTRLSRTIGYTVKKT    60
Po/PCV2/DOM/GES7/2020      MTYPRRRFRRRRHRPRSHLGQILRRRPWLVHPRHRYRWRRKNGIFNTRLSRTIGYTVKKT    60
Po/PCV2/DOM/MA5/2020       MTYPRRRFRRRRHRPRSHLGQILRRRPWLVHPRHRYRWRRKNGIFNTRLSRTIGYTVKKT    60
Po/PCV2/DOM/M8/2020        MTYPRRRFRRRRHRPRSHLGQILRRRPWLVHPRHRYRWRRKNGIFNTRLSRTIGYTVKKT    60
Po/PCV2/DOM/MA9/2020       MTYPRRRFRRRRHRPRSHLGQILRRRPWLVHPRHRYRWRRKNGIFNTRLSRTIGYTVKKT    60
Po/PCV2/DOM/N8/2020        MTYPRRRFRRRRHRPRSHLGQILRRRPWLVHPRHRYRWRRKNGIFNTRLSRTIGYTVKKT    60
Po/PCV2/DOM/ENG5/2020      MTYPRRRFRRRRHRPRSHLGQILRRRPWLVHPRHRYRWRRKNGIFNTRLSRTIGYTVKKT    60
Po/PCV2/DOM/P4/2021        MTYPRRRFRRRRHRPRSHLGQILRRRPWLVHPRHRYRWRRKNGIFNTRLSRTIGYTVKKT    60
Po/PCV2/DOM/Z11/2021       MTYPRRRFRRRRHRPRSHLGQILRRRPWLVHPRHRYRWRRKNGIFNTRLSRTIGYTVKKT    60
Po/PCV2/DOM/Z13/2021       MTYPRRRFRRRRHRPRSHLGQILRRRPWLVHPRHRYRWRRKNGIFNTRLSRTIGYTVKKT    60
Po/PCV2a/HQ202949          MTYPRRRYRRRRHRPRSHLGQILRRRPWLVHPRHRYRWRRKNGIFNTRLSRTFGYTVKRT    60
Po/PCV2b/KY806003          MTYPRRRYRRRRHRPRSHLGQILRRRPWLVHPHHRYRWRRKNGIFNTRLSRTFGYTIKRT    60
Po/PCV2c/EU148503          MTYPRRRYRRRRHRPRSHLGHILRRRPWLVHPRHRYRWRRKNGIFNARLSRSFVYTVNAS    60
Po/PCV2d/MH323413          MTYPRRRFRRRRHRPRSHLGQILRRRPWLVHPRHRYRWRRKNGIFNTRLSRTIGYTVKKT    60
Po/PCV2d/JX535296          MTYPRRRFRRRRHRPRSHLGQILRRRPWLVHPRHRYRWRRKNGIFNTRLSRTIGYTVKKT    60
Po/PCV2d/MF616427          MTYPRRRFRRRRHRPRSHLGQILRRRPWLVHPRHRYRWRRKNGIFNTRLSRTIGYTVKKT    60
Po/PCV2d/MF142267          MTYPRRRFRRRRHRPRSHLGQILRRRPWLVHPRHRYRWRRKNGIFNTRLSRTIGYTVKKT    60
Po/PCV2e/KT870147          MTYPRRRYRRRRHRPRSHLGQILRRRPWLVHPRHRYRWRRKNGIFNARLSRSFVYTVVTS    60
Po/PCV2f/LC008137          MTYPRRRYRRRRHRPRSHLGHILRRRPWLVHPRHRYRWRRKNGIFNARLSRTFGYTVKAS    60
Po/PCV2g/FJ998185          MTYPRRRYRRRRHRPRSHLGQILRRRPWLVHPRHRYRWRRKNGIFNTRLSRTFGYTVARS    60
Po/PCV2h/JX506730          MTYPRRRFRRRRHRPRSHLGQILRRRPWLVHPRHRYRWRRKNGIFNTRLSRTFGYTVKKT    60
                           ******* ************ *********** ************* ****   **    

Po/PCV2/DOM/DE7/2020       TVRTPSWNVDMMRFNINDFLPPGGGSNPLTVPFEYYRIRKVKVEFWPCSPITQGDRGVGS    120
Po/PCV2/DOM/VE22/2020      TVRTPSWNVDMMRFNINDFLPPGGGSNPLTVPFEYYRIRKVKVEFWPCSPITQGDRGVGS    120
Po/PCV2/DOM/GES15/2020     TVRTPSWNVDMMRFNINDFLPPGGGSNPLTVPFEYYRIRKVKVEFWPCSPITQGDRGVGS    120
Po/PCV2/DOM/ENG22/2020     TVRTPSWNVDMMRFNINDFLPPGGGSNPLTVPFEYYRIRKVKVEFWPCSPITQGDRGVGS    120
Po/PCV2/DOM/DE92/2020      TVRTPSWNVDMMRFNINDFLPPGGGSNPLTVPFEYYRIRKVKVEFWPCSPITQGDRGVGS    120
Po/PCV2/DOM/DE102/2020     TVRTPSWNVDMMRFNINDFLPPGGGSNPLTVPFEYYRIRKVKVEFWPCSPITQGDRGVGS    120
Po/PCV2/DOM/ENG52/2020     TVRTPSWNVDMMRFNINDFLPPGGGSNPLTVPFEYYRIRKVKVEFWPCSPITQGDRGVGS    120
Po/PCV2/DOM/GE2/2020       TVRTPSWNVDMMRFNINDFLPPGGGSNPLTVPFEYYRIRKVKVEFWPCSPITQGDRGVGS    120
Po/PCV2/DOM/GES7/2020      TVRTPSWNVDMMRFNINDFLPPGGGSNPLTVPFEYYRIRKVKVEFWPCSPITQGDRGVGS    120
Po/PCV2/DOM/MA5/2020       TVRTPSWNVDMMRFNINDFLPPGGGSNPLTVPFEYYRIRKVKVEFWPCSPITQGDRGVGS    120
Po/PCV2/DOM/M8/2020        TVRTPSWNVDMMRFNINDFLPPGGGSNPLTVPFEYYRIRKVKVEFWPCSPITQGDRGVGS    120
Po/PCV2/DOM/MA9/2020       TVRTPSWNVDMMRFNINDFLPPGGGSNPLTVPFEYYRIRKVKVEFWPCSPITQGDRGVGS    120
Po/PCV2/DOM/N8/2020        TVRTPSWNVDMMRFNINDFLPPGGGSNPLTVPFEYYRIRKVKVEFWPCSPITQGDRGVGS    120
Po/PCV2/DOM/ENG5/2020      TVRTPSWNVDMMRFNINDFLPPGGGSNPLTVPFEYYRIRKVKVEFWPCSPITQGDRGVGS    120
Po/PCV2/DOM/P4/2021        TVRTPSWNVDMMRFNINDFLPPGGGSNPLTVPFEYYRIRKVKVEFWPCSPITQGDRGVGS    120
Po/PCV2/DOM/Z11/2021       TVRTPSWNVDMMRFNINDFLPPGGGSNPLTVPFEYYRIRKVKVEFWPCSPITQGDRGVGS    120
Po/PCV2/DOM/Z13/2021       TVRTPSWNVDMMRFNINDFLPPGGGSNPLTVPFEYYRIRKVKVEFWPCSPITQGDRGVGS    120
Po/PCV2a/HQ202949          TVTTPSWAVDMMRFKLDDFVPPGGGTNKISIPFEYYRIRKVKVEFWPCSPITQGDRGVGS    120
Po/PCV2b/KY806003          TVKTPSWAVDMMRFNINDFLPPGGGSNPRSVPFEYYRIRKVKVEFWPCSPITQGDRGVGS    120
Po/PCV2c/EU148503          QVSPPSWAVDMMRFNINQFLPPGGGSNPLTVPFEYYRIRKVKVEFFARSPITQGDRGVGS    120
Po/PCV2d/MH323413          TVRTPSWNVDMMRFNINDFLPPGGGSNPLTVPFEYYRIRKAKVEFWPCSPITQGDRGVGS    120
Po/PCV2d/JX535296          TVRTPSWNVDMMRFNINDFLPPGGGSNPLTVPFEYYRIRKVKVEFWPCSPITQGDRGVGS    120
Po/PCV2d/MF616427          TVRTPSWNVDMMRFNINDFLPPGGGSNPLTVPFEYYRIRKVKVEFWPCSPITQGDRGVGS    120
Po/PCV2d/MF142267          TVRTPSWNVDMMRFNINDFLPPGGGSNPLTVPFEYYRIRKVKVEFWPCSPITQGDRGVGS    120
Po/PCV2e/KT870147          TVTPPSWAVDMMRFNIGDFLPPGGGTNPLSVPFEYYRIRKVKVEFFARSPITQGERGVGS    120
Po/PCV2f/LC008137          TVTTPSWAVDMLRFNINDFLPPGGGSNPLTVPFEYYRIRKVKVEFWPCSPITQGDRGVGS    120
Po/PCV2g/FJ998185          TVSTPSWAVDMMRFNINDFLPPGGGSNPLTVPFEYYRIRKVKVEFWPCSPITQGDRGVGS    120
Po/PCV2h/JX506730          TVRTPSWAVDMMRFNINDFLPPGGGSNPLTVPFEYYRIRKVKVEFWPCSPITQGDRGVGS    120
                            *  *** *** **    * ***** *    ********* ****   ****** *****
```

```
Po/PCV2/DOM/DE7/2020        TAVILDDNFVTKANALTYDPYVNYSSRHTITQPFSYHSRYFTPKPVLDGTIDYFQPNNKR    180
Po/PCV2/DOM/VE22/2020       TAVILDDNFVTKANALTYDPYVNYSSRHTITQPFSYHSRYFTPKPVLDGTIDYFQPNNKR    180
Po/PCV2/DOM/GES15/2020      TAVILDDNFVTKANALTYDPYVNYSSRHTITQPFSYHSRYFTPKPVLDRTIDYFQPNNKR    180
Po/PCV2/DOM/ENG22/2020      TAVILDDNFVTKANALTYDPYVNYSSRHTITQPFSYHSRYFTPKPVLDGTIDYFQPNNKR    180
Po/PCV2/DOM/DE92/2020       TAVILDDNFVTKANALTYDPYVNYSSRHTITQPFSYHSRYFTPKPVLDGTIDYFQPNNKR    180
Po/PCV2/DOM/DE102/2020      TAVILDDNFVTKANALTYDPYVNYSSRHTITQPFSYHSRYFTPKPVLDRAIDYFQPNNKR    180
Po/PCV2/DOM/ENG52/2020      TAVILDDNFVTKANALTYDPYVNYSSRHTITQPFSYHSRYFTPKPVLDGTIDYFQPNNKR    180
Po/PCV2/DOM/GE2/2020        TAVILDDNFVTKANALTYDPYVNYSSRHTITQPFSYHSRYFTPKPVLDGTIDYFQPNNKR    180
Po/PCV2/DOM/GES7/2020       TAVILDDNFVTKANALTYDPYVNYSSRHTITQPFSYHSRYFTPKPVLDGTIDYFQPNNKR    180
Po/PCV2/DOM/MA5/2020        TAVILDDNFVTKANALTYDPYVNYSSRHTITQPFSYHSRYFTPKPVLDGTIDYFQPNNKR    180
Po/PCV2/DOM/M8/2020         TAVILDDNFVTKANALTYDPYVNYSSRHTITQPFSYHSRYFTPKPVLDGTIDYFQPNNKR    180
Po/PCV2/DOM/MA9/2020        TAVILDDNFVTKANALTYDPYVNYSSRHTITQPFSYHSRYFTPKPVLDGTIDYFQPNNKR    180
Po/PCV2/DOM/N8/2020         TAVILDDNFVTKANALTYDPYVNYSSRHTITQPFSYHSRYFTPKPVLDGTIDYFQPNNKR    180
Po/PCV2/DOM/ENG5/2020       TAVILDDNFVTKANALTYDPYVNYSSRHTITQPFSYHSRYFTPKPVLDGTIDYFQPNNKR    180
Po/PCV2/DOM/P4/2021         TAVILDDNFVTKANALTYDPYVNYSSRHTITQPFSYHSRYFTPKPVLDGTIDYFQPNNKR    180
Po/PCV2/DOM/Z11/2021        TAVILDDNFVTKANALTYDPYVNYSSRHTITQPFSYHSRYFTPKPVLDRTIDYFQPNNKR    180
Po/PCV2/DOM/Z13/2021        TAVILDDNFVTKANALTYDPYVNYSSRHTITQPFSYHSRYFTPKPVLDRTIDYFQPNNKR    180
Po/PCV2a/HQ202949           TAVILDDNFVPKANALTYDPYVNYSSRHTIPQPFSYHSRYFTPKPVLDSTIDYFQPNNKR    180
Po/PCV2b/KY806003           SAVILDDNFVTKATALTYDPYVNYSSRHTITQPFSYHSRYFTPKPVLDSTIDYFQPNNKR    180
Po/PCV2c/EU148503           TAVILNDNFVTKATALTYDPYVNYSSRHTITQPFSYHSRYFTPKPVLDSTIDYFQPNNKR    180
Po/PCV2d/MH323413           TAVILDDNFVTKANALTYDPYVNYSSRHTITQPFSYHSRYFTPKPVLDRAIDYFQPNNKR    180
Po/PCV2d/JX535296           TAVILDDNFVTKANALTYDPYVNYSSRHTITQPFSYHSRYFTPKPVLDRTIDYFQPNNKR    180
Po/PCV2d/MF616427           TAVILDDNFVTKANALTYDPYVNYSSRHTITQPFSYHSRYFTPKPVLDRTIDYFQPNNKR    180
Po/PCV2d/MF142267           TAVILDDNFVTKANALTYDPYVNYSSRHTITRPFSYHSRYFTPKPVLDGTIDYFQPNNKR    180
Po/PCV2e/KT870147           SAVILDDNFVNKTNNLSYDPYVNYSSRHTITQPFSYHSRYFTPKPVLDRIIDYFQPNNKR    180
Po/PCV2f/LC008137           SAVILDDNFVTKATALTYDPYVNYSSRHTITQPFSYHSRYFTPEPVLDSTLDYFQPNNKR    180
Po/PCV2g/FJ998185           TAVILDDNFVTKATALTYDPYVNYSSRHTIPQPFSYHSRYFTPKPVLDRTIDYFQPNNKR    180
Po/PCV2h/JX506730           TAVILDDNFVPKATALTYDPYVNYSSRHTITQPFSYHSRYFTPKPVLDSTIDYFQPNNKR    180
                             **** **** *   * *************  *********** ****   *********

Po/PCV2/DOM/DE7/2020        NQLWLRLQTTGNVDHVGLGTAFENSIYDQDYNIRITMYVQFREFNLKDPPLNPK----      234
Po/PCV2/DOM/VE22/2020       NQLWLRLQTTGNVDHVGLGTAFENSIYDQDYNIRITMYVQFREFNLKDPPLNPK----      234
Po/PCV2/DOM/GES15/2020      NQLWLRLQTTGNVDHVGLGTAFENSIYDQDYNIRITMYVQFREFNLKDPPLNPK----      234
Po/PCV2/DOM/ENG22/2020      NQLWLRLQTTGNVDHVGLGTAFENSIYDQDYNIRITMYVQFREFNLKDPPLNPK----      234
Po/PCV2/DOM/DE92/2020       NQLWLRLQTTGNVDHVGLGTAFENSIYDQDYNIRITMYVQFREFNLKDPPLNPK----      234
Po/PCV2/DOM/DE102/2020      NQLWLRLQTTGNVDHVGLGTAFENSIYDQDYNIRITMYVQFREFNLKDPPLNPK----      234
Po/PCV2/DOM/ENG52/2020      NQLWLRLQTTGNVDHVGLGTAFENSIYDQDYNIRITMYVQFREFNLKDPPLNPK----      234
Po/PCV2/DOM/GE2/2020        NQLWLRLQTTGNVDHVGLGTAFENSIYDQDYNIRITMYVQFREFNLKDPPLNPK----      234
Po/PCV2/DOM/GES7/2020       NQLWLRLQTTGNVDHVGLGTAFENSIYDQDYNIRITMYVQFREFNLKDPPLNPK----      234
Po/PCV2/DOM/MA5/2020        NQLWLRLQTTGNVDHVGLGTAFENSIYDQDYNIRITMYVQFREFNLKDPPLNPK----      234
Po/PCV2/DOM/M8/2020         NQLWLRLQTTGNVDHVGLGTAFENSIYDQDYNIRITMYVQFREFNLKDPPLNPK----      234
Po/PCV2/DOM/MA9/2020        NQLWLRLQTTGNVDHVGLGTAFENSIYDQDYNIRITMYVQFREFNLKDPPLNPK----      234
Po/PCV2/DOM/N8/2020         NQLWLRLQTTGNVDHVGLGTAFENSIYDQDYNIRITMYVQFREFNLKDPPLNPK----      234
Po/PCV2/DOM/ENG5/2020       NQLWLRLQTTGNVDHVGLGTAFENSIYDQDYNIRITMYVQFREFNLKDPPLNPK----      234
Po/PCV2/DOM/P4/2021         NQLWLRLQTTGNVDHVGLGTAFENSIYDQDYNIRITMYVQFREFNLKDPPLNPK----      234
Po/PCV2/DOM/Z11/2021        NQLWLRLQTTGNVDHVGLGTAFENSIYDQDYNIRITMYVQFREFNLKDPPLNPK----      234
Po/PCV2/DOM/Z13/2021        NQLWLRLQTTGNVDHVGLGTAFENSIYDQDYNIRITMYVQFREFNLKDPPLNPK----      234
Po/PCV2a/HQ202949           NQLWLRLQTSRNVDHVGLGTAFENSKYDQDYNIRVTMYVQFREFNLKDPPLNP-----      233
Po/PCV2b/KY806003           NQLWLRLQTAGNVDHVGLGTAFENSIYDQEYNIRVTMYVQFREFNLKDPPLKP-----      233
Po/PCV2c/EU148503           NQLWMRLQTTGNVDHVGLGHAFQNSTNAQAYNVRVTMYVQFREFNLKDPPLNPK----      234
Po/PCV2d/MH323413           NQLWLRLQTTGNVDHVGLGTAFENSIYDQDYNIRITMYVQFREFNLKDPPLNPK----      234
Po/PCV2d/JX535296           NQLWLRLQTTGNVDHVGLGTAFENSIYDQDYNIRITMYVQFREFNLKDPPLNPK----      234
Po/PCV2d/MF616427           NQLWLRLQTTGNVDHVGLGTAFENSIYDQDYNIRITMYVQFREFNLKDPPLNPK----      234
Po/PCV2d/MF142267           NQLWLRLQTTGNVDHVGLGTAFENSIYDQDYNIRITMYVQFREFNLKDPPLNPK----      234
Po/PCV2e/KT870147           NQLWLRLQTSGNVDHTGLGTAFQHSTTAQAYNVRVTLYVQFREFNMKDPPLPSPLSYM      238
Po/PCV2f/LC008137           NQLWMRLQTTGNVDHVGLGTAFENSKYDQDYNVRITVYVQFREFNLKDPPLNTK----      234
Po/PCV2g/FJ998185           NQLWLRLQTSGNVDHVGLGTAFENSKYDQDYNIRITMYVQFREFNLKDPPLNPK----      234
Po/PCV2h/JX506730           NQLWLRLQTSANVDHVGLGTAFENSIYDQDYNIRVTMYVQFREFNLKDPPLHPK----      234
                            **** ****  **** *** **  *   * ** * * ******** *****
```
