# Supplementary material for: Detection and Complete Genome Analysis of Porcine Circovirus 2 (PCV2) and an Unclassified CRESS DNA Virus from Diarrheic Pigs in the Dominican Republic: First Evidence for Predominance of PCV2d from the Caribbean Region
Source: Viruses. 2022 Aug 17;14(8):1799. doi: 10.3390/v14081799 (PMC9415081; doi:10.3390/v14081799)
Supplement: Supplementary file 1 [file viruses-14-01799-s001.zip › Supplementary material S6.pdf]

Supplementary material S6. Multiple alignment of the putative replication-associated proteins (Rep) of porcine circovirus 2 (PCV2) strains from the Dominican Republic with those of virus strains representing the 8 PCV2 genotypes (PCV2a-h). Unique/rare amino acid (aa) mismatches in the putative Rep of PCV2 strains from the Dominican Republic are shown with green. The other aa mismatches have been highlighted with blue. The host/virus species/country/virus name/year are shown for the PCV2 strains from the Dominican Republic, whilst the host/PCV2 genotype/GenBank accession number have been mentioned for the reference PCV2 strains. A '*' denotes an identical aa residue, whilst '-' indicates absence of an aa residue. Numbers to the right indicate the positions of the aa for respective PCV2 strains.

```
Po/PCV2/DOM/DE7/2020       MPSKKNGRSGPQPHKRWVFTLNNPSEDERKKIRELPISLFDYFIVGEEGNEEGRTPHLQG    60
Po/PCV2/DOM/VE22/2020      MPSKKNGRSGPQPHKRWVFTLNNPSEDERKKIRELPISLFDYFIVGEEGNEEGRTPHLQG    60
Po/PCV2/DOM/GES15/2020     MPSKKNGRSGPQPHKRWVFTLNNPSKDERKKIRELPISLFDYFIVGEEGNEEGRTPHLQG    60
Po/PCV2/DOM/ENG22/2020     MPSKKNGRSGPQPHKRWVFTLNNPSEDERKKIRELPISLFDYFIVGEEGNEEGRTPHLQG    60
Po/PCV2/DOM/DE92/2020      MPSKKNGRSGPQPHKRWVFTLNNPSEDERKKIRELPISLFDYFIVGEEGNEEGRTPHPQG    60
Po/PCV2/DOM/DE102/2020     MPSKKNGRSGPQPHKRWVFTLNNPSEDERKKIRELPISLFDYFIVGEEGNEEGRTPHLQG    60
Po/PCV2/DOM/ENG52/2020     MPSKKNGRSGPQPHKRWVFTLNNPSEDERKKIRELPISLFDYFIVGEEGNEEGRTPHLQG    60
Po/PCV2/DOM/GE2/2020       MPSKKNGRSGPQPHKRWVFTLNNPSEDERKKIRELPISLFDYFIVGEEGNEEGRTPHLQG    60
Po/PCV2/DOM/GES7/2020      MPSKKNGRSGPQPHKRWVFTLNNPSEDERKKIRELPISLFDYFIVGEEGNEEGRTPHLQG    60
Po/PCV2/DOM/MA5/2020       MPSKKNGRSGPQPHKRWVFTLNNPSEDERKKIRELPISLFDYFIVGEEGNEEGRTPHLQG    60
Po/PCV2/DOM/M8/2020        MPSKKNGRSGPQPHKRWVFTLNNPSEDERKKIRELPISLFDYFIVGEEGNEEGRTPHLQG    60
Po/PCV2/DOM/MA9/2020       MPSKKNGRSGPQPHKRWVFTLNNPSEDERKKIRELPISLFDYFIVGEEGNEEGRTPHLQG    60
Po/PCV2/DOM/N8/2020        MPSKKNGRSGPQPHKRWVFTLNNPSEDERKKIRELPISLFDYFIVGEEGNEEGRTPHLQG    60
Po/PCV2/DOM/ENG5/2020      MPSKKNGRSGPQPHKRWVFTLNNPSEDERKKIRELPISLFDYFIVGEEGNEEGRTPHLQG    60
Po/PCV2/DOM/P4/2021        MPSKKNGRSGPQPHKRWVFTLNNPSEDERKKIRELPISLFDYFIVGEEGNEEGRTPHLQG    60
Po/PCV2/DOM/Z11/2021       MPSKKNGRSGPQPHKRWVFTLNNPSEDERKKIRELPISLFDYFIVGEEGNEEGRTPQLQG    60
Po/PCV2/DOM/Z13/2021       MPSKKNGRSGPQPHKRWVFTLNNPSEDERKKIRELPISLFDYFIVGEEGNEEGRTPHLQG    60
Po/PCV2a/HQ202949          MPSKKNGRSGPQPHKRWVFTLNNPSEDERKKIRELPISLFDYFIVGEEGNEEGRTPHLQG    60
Po/PCV2b/DQ220736          MPSKKNGRSGPQPHKRWVFTLNNPSEDERKKIRDLPISLFDYFIVGEEGNEEGRTPHLQG    60
Po/PCV2c/EU148503          MPSKKNGRSGPQPHKRWVFTLNNPSEDERKKIRELPISLFDYFIVGEEGNEEGRTPHLQG    60
Po/PCV2d/MH323413          MPSKKNGRSGPQPHKRWVFTLNNPSEDERKKIRELPISLFDYFIVGEEGNEEGRTPHLQG    60
Po/PCV2d/JX535296          MPSKKSGRSGPQPHKRWVFTLNNPSEDERKKIRELPISLFDYFIVGEEGNEEGRTPHLQG    60
Po/PCV2d/MF616427          MPSKKSGRSGPQPHKRWVFTLNNPSEDERKKIRELPISLFDYFIVGEEGNEEGRTPHLQG    60
Po/PCV2d/MF142267          MPSKKSGRSGPQPHKRWVFTLNNPSEDERKKIRELPISLFDYFIVGEEGNEEGRTPHLQG    60
Po/PCV2d/KX831483          MPSKKSGRSGPQPHKRWVFTLNNPSEDERKKIRELPISLFDYFIVGEEGNEEGRTPHLQG    60
Po/PCV2e/KT870147          MPSKKNGRSGPQPHKRWVFTLNNPSEDERKKIRELPISLFDYFIVGEEGNEEGRTPHLQG    60
Po/PCV2f/LC008137          MPSKKNGRSGPQPHKRWVFTLNNPSEDERKKIRELPISLFDYFIVGEEGNEEGRTPHLQG    60
Po/PCV2g/FJ998185          MPSKKNGRSGPQPYKRWVFTLNNPSEDERKKIRELPISLFDYFIVGEEGNEEGRTPHLQG    60
Po/PCV2h/JX506730          MPSKKNGRSGPQPHKRWVFTLNNPSEDERKKIRELPISLFDYFIVGEEGNEEGRTPHLQG    60
                           ***** ******* *********** ******* **********************  **

Po/PCV2/DOM/DE7/2020       FANFVKKQTFNKVKWYLGARCHIEKAKGTDQQNKEYCSKEGNLLIECGAPRSQGQRSDLS    120
Po/PCV2/DOM/VE22/2020      FANFVKKQTFNKVKWYLGARCHIEKAKGTDQQNKEYCSKEGNLLIECGAPRSQGQRSDLS    120
Po/PCV2/DOM/GES15/2020     FANFVKKQTFNKVKWYLGARCHIEKAKGTDQQNKEYCSKEGNLLIECGAPRSQGQRSDLS    120
Po/PCV2/DOM/ENG22/2020     FANFVKKQTFNKVKWYLGARCHIEKAKGTDQQNKEYCSKEGNLLIECGAPRSQGQRSDLS    120
Po/PCV2/DOM/DE92/2020      FANFVKKQTFNKVKWYLGARCHIEKAKGTDQQNKEYCSKEGNLLIECGAPRSQGQRSDLS    120
Po/PCV2/DOM/DE102/2020     FANFVKKQTFNKVKWYLGARCHIEKAKGTDQQNKEYCSKEGNLLIECGAPRSQGQRSDLS    120
Po/PCV2/DOM/ENG52/2020     FANFVKKQTFNKVKWYLGARCHIEKAKGTDQQNKEYCSKEGNLLIECGAPRSQGQRSDLS    120
Po/PCV2/DOM/GE2/2020       FANFVKKQTFNKVKWYLGARCHIEKAKGTDQQNKEYCSKEGNLLIECGAPRSQGQRSDLS    120
Po/PCV2/DOM/GES7/2020      FANFVKKQTFNKVKWYLGARCHIEKAKGTDQQNKEYCSKEGNLLIECGAPRSQGQRSDLS    120
Po/PCV2/DOM/MA5/2020       FANFVKKQTFNKVKWYLGARCHIEKAKGTDQQNKEYCSKEGNLLIECGAPRSQGQRSDLS    120
Po/PCV2/DOM/M8/2020        FANFVKKQTFNKVKWYLGARCHIEKAKGTDQQNKEYCSKEGNLLIECGAPRSQGQRSDLS    120
Po/PCV2/DOM/MA9/2020       FANFVKKQTFNKVKWYLGARCHIEKAKGTDQQNKEYCSKEGNLLIECGAPRSQGQRSDLS    120
Po/PCV2/DOM/N8/2020        FANFVKKQTFNKVKWYLGARCHIEKAKGTDQQNKEYCSKEGNLLIECGAPRSQGQRSDLS    120
Po/PCV2/DOM/ENG5/2020      FANFVKKQTFNKVKWYLGARCHIEKAKGTDQQNKEYCSKEGNLLIECGAPRSQGQRSDLS    120
Po/PCV2/DOM/P4/2021        FANFVKKQTFNKVKWYLGVRCHIEKAKGTDQQNKEYCSKEGNLLIECGAPRSQGQRSDLS    120
Po/PCV2/DOM/Z11/2021       FANFVKKQTFNKVKWYLGARCHIEKAKGTDQQNKEYCSKEGNLLIECGAPRSQGQRSDLS    120
Po/PCV2/DOM/Z13/2021       FANFVKKQTFNKVKWYLGARCHIEKAKGTDQQNKEYCSKEGNLLIECGAPRSQGQRSDLS    120
Po/PCV2a/HQ202949          FANFVKKQTFNKVKWYLGARCHIEKAKGTDQQNKEYCSKEGNLLIECGAPRSQGQRSDLS    120
Po/PCV2b/DQ220736          FANFVKKQTFNKVKWYLGARCHIEKAKGTDQQNKEYCSKEGNLLIECGAPRSQGQRSDLS    120
Po/PCV2c/EU148503          FANFVKKKTFNKVKWYLGARCHIEKAKGTDQQNKEYCSKEGNLLMECGAPRSQGQRSDLS    120
Po/PCV2d/MH323413          FANFVKKQTFNKVKWYFGARCHIEKAKGTDQQNKEYCSKEGNLLIECGAPRSQGQRSDLS    120
Po/PCV2d/JX535296          FANFVKKQTFNKVKWYFGARCHIEKAKGTDQQNKEYCSKEGNLLIECGAPRSQGQRSDLS    120
Po/PCV2d/MF616427          FANFVKKQTFNKVKWYFGARCHIEKAKGTDQQNKEYCSKEGNLLIECGAPRSQGQRSDLS    120
Po/PCV2d/MF142267          FANFVKKQTFNKVKWYFGARCHIEKAKGTDQQNKEYCSKEGNLLIECGAPRSQGYRSDLS    120
Po/PCV2d/KX831483          FANFVKKQTFNKVKWYLGARCHIEKAKGTDQQNKEYCSKEGNLLIECGAPRSQGQRSDLS    120
Po/PCV2e/KT870147          FANFVKKQTFNKVKWYFGARCHIEKAKGTDHQNKEYCSKEGNLLIECGAPRSQGQRSDLS    120
Po/PCV2f/LC008137          FANFVKKQTFNKVKWYFGARCHIEKAKGTDQQNKEYCSKEGNLLIECGASRSQGQRSDLS    120
Po/PCV2g/FJ998185          FANFVKKQTFNKVKWYFGARCHIEKAKGTDQQNKEYCSKEGNLLMECGAPRSQGQRSDLS    120
Po/PCV2h/JX506730          FANFVKKQTFNKVKWYFGARCHIEKAKGTDQQNKEYCSKEGNLLMECGAPRSQGQRSDLS    120
                           ******* ******** * *********** ************* **** **** *****
```

```
Po/PCV2/DOM/DE7/2020        TAVSTLLESGSLVTVAEQHPVTFVRNFRGLAELLKVSGKMQKRDWKTNVHVIVGPPGCGK    180
Po/PCV2/DOM/VE22/2020       TAVSTLLESGSLVTVAEQHPVTFVRNFRGLAELLKVSGKMQKRDWKTNVHVIVGPPGCGK    180
Po/PCV2/DOM/GES15/2020      TAVSTLLESGSLVTVAEQHPVTFVRNFRGLAELLKVSGKMQKRDWKTNVHVIVGPPGCGK    180
Po/PCV2/DOM/ENG22/2020      TAVSTLLESGSLVTVAEQHPVTFVRNFRGLAELLKVSGKMQKRDWKTNVHVIVGPPGCGK    180
Po/PCV2/DOM/DE92/2020       TAVSTLLESGSLVTVAEQHPVTFVRNFRGLAELLKVSGKMQKRDWKTNVHVIVGPPGCGK    180
Po/PCV2/DOM/DE102/2020      TAVSTLLESGSLVTVAEQHPVTFVRNFRGLAELLKVSGKMQKRDWKTNVHVIVGPPGCGK    180
Po/PCV2/DOM/ENG52/2020      TAVSTLLESGSLVTVAEQHPVTFVRNFRGLAELLKVSGKMQKRDWKTNVHVIVGPPGCGK    180
Po/PCV2/DOM/GE2/2020        TAVSTLLESGSLVTVAEQHPVTFVRNFRGLAELLKVSGKMQKRDWKTNVHVIVGPPGCGK    180
Po/PCV2/DOM/GES7/2020       TAVSTLLESGSLVTVAEQHPVTFVRNFRGLAELLKVSGKMQKRDWKTNVHVIVGPPGCGK    180
Po/PCV2/DOM/MA5/2020        TAVSTLLESGSLVTVAEQHPVTFVRNFRGLAELLKVSGKMQKRDWKTNVHVIVGPPGCGK    180
Po/PCV2/DOM/M8/2020         TAVSTLLESGSLVTVAEQHPVTFVRNFRGLAELLKVSGKMQKRDWKTNVHVIVGPPGCGK    180
Po/PCV2/DOM/MA9/2020        TAVSTLLESGSLVTVAEQHPVTFVRNFRGLAELLKVSGKMQKRDWKTNVHVIVGPPGCGK    180
Po/PCV2/DOM/N8/2020         TAVSTLLESGSLVTVAEQHPVTFVRNFRGLAELLKVSGKMQKRDWKTNVHVIVGPPGCGK    180
Po/PCV2/DOM/ENG5/2020       TAVSTLLESGSLVTVAEQHPVTFVRNFRGLAELLKVSGKMQKRDWKTNVHVIVGPPGCGK    180
Po/PCV2/DOM/P4/2021         TAVSTLLESGSLVTVAEQHPVTFVRNFRGLAELLKVSGKMQKRDWKTNVHVIVGPPGCGK    180
Po/PCV2/DOM/Z11/2021        TAVSTLLESGSLVTVAEQHPVTFVRNFRGLAELLKVSGKMQKRDWKTNVHVIVGPPGCGK    180
Po/PCV2/DOM/Z13/2021        TAVSTLLESGSLVTVAEQHPVTFVRNFRGLAELLKVSGKMQKRDWKTNVHVIVGPPGCGK    180
Po/PCV2a/HQ202949           TAVSTLLESGSLVTVAEQHPVTFVRNFRGLAELLKVSGKMQKRDWKTNVHVIVGPPGCGK    180
Po/PCV2b/DQ220736           TAVSTLLESGSLVTVAEQYPVTFVRNFRGLAELLKVSGKMQKRDWKTNVHVIVGPPGCGK    180
Po/PCV2c/EU148503           TAVSTLLESGSLVTVAEQHPVTFVRNFRGLAELLKVSGKMQKRDWKTNVHVIVGPPGCGK    180
Po/PCV2d/MH323413           TAVSTLLESGSLVTVAEQHPVTFVRNFRGLAELLKVSGKMQKRDWKTNVHVIVGPPGCGK    180
Po/PCV2d/JX535296           TAVSTLLESGSLVTVAEQHPVTFVRNFRGLAELLKVSGKMQKRDWKTNVHVIVGPPGCGK    180
Po/PCV2d/MF616427           TAVSTLLESGSLVTVAEQHPVTFVRNFRGLAELLKVSGKMQKRDWKTNVHVIVGPPGCGK    180
Po/PCV2d/MF142267           TAVSTLLESGSLVTVAEQHPVTFVRNFRGLAELLKVSGKMQKRDWKTNVHVIVGPPGCGK    180
Po/PCV2d/KX831483           TAVSTLLESGSLVTVAEQHPVTFVRNFRGLAELLKVSGKMQKRDWKTNVHVIVGPPGCGK    180
Po/PCV2e/KT870147           TAVSTLLESGSLVTVAEQHPVTFVRNFRGLAELLKVSGKMQKRDWKTNVHVIVGPPGCGK    180
Po/PCV2f/LC008137           TAVSTLLESGSLVTVAEQHPVTFVRNFRGLAELLKVSGKMQKRDWKTNVHVIVGPPGCGK    180
Po/PCV2g/FJ998185           TAVSTLLESGSLVTVAEQYPVTFVRNFRGLAELLKVSGKMQKRDWKTNVHVIVGPPGCGK    180
Po/PCV2h/JX506730           TAVSTLLESGSLVTAAEQHPVTFVRNFRGLAELLKVSGKMQKRDWKTNVHVIVGPPGCGK    180
                            ************** *** *****************************************

Po/PCV2/DOM/DE7/2020        SKWAANFADPETTYWKPPRNKWWDGYHGEEVVVIDDFYGWLPWDDLLRLCDRYPLTVETK    240
Po/PCV2/DOM/VE22/2020       SKWAANFADPETTYWKPPRNKWWDGYHGEEVVVIDDFYGCLPWDDLLRLCDPYPLTVETK    240
Po/PCV2/DOM/GES15/2020      SKWAANFADPETTYWKPPRNKWWDGYHGEEVVVIDDFYGWLPWDDLLRLCDRYPLTVETK    240
Po/PCV2/DOM/ENG22/2020      SKWAANFADPETTYWKPPRNKWWDGYHGEEVVVIDDFYGWLPWDDLLRLCDRYPLTVETK    240
Po/PCV2/DOM/DE92/2020       SKWAANFADPETTYWKPPRNKWWDGYHGEEVVVIDDFYGWLPWDDLLRLCDRYPLTVETK    240
Po/PCV2/DOM/DE102/2020      SKWAANFADPETTYWKPPRNKWWDGYHGEEVVVIDDFYGWLPWDDLLRLCDRYPLTVETK    240
Po/PCV2/DOM/ENG52/2020      SKWAANFADPETTYWKPPRNKWWDGYHGEEVVVIDDFYGWLPWDDLLRLCDRYPLTVETK    240
Po/PCV2/DOM/GE2/2020        SKWAANFADPETTYWKPPRNKWWDGYHGEEVVVIDDFYGWLPWDDLLRLCDRYPLTVETK    240
Po/PCV2/DOM/GES7/2020       SKWAANFADPETTYWKPPRNKWWDGYHGEEVVVIDDFYGWLPWDDLLRLCDRYPLTVETK    240
Po/PCV2/DOM/MA5/2020        SKWAANFADPETTYWKPPRNKWWDGYHGEEVVVIDDFYGWLPWDDLLRLCDRSPLTVENK    240
Po/PCV2/DOM/M8/2020         SKWAANFADPETTYWKPPRNKWWDGYHGEEVVVIDDFYGWLPWDDLLRLCDRYPLTVETK    240
Po/PCV2/DOM/MA9/2020        SKWAANFADPETTYWKPPRNKWWDGYHGEEVVVIDDFYGWLPWDDLLRLCDRYPLTVETK    240
Po/PCV2/DOM/N8/2020         SKWAANFADPETTYWKPPRNKWWDGYHGEEVVVIDDFYGWLPWDDLLRLCDRYPLTVETK    240
Po/PCV2/DOM/ENG5/2020       SKWAANFADPETTYWKPPRNKWWDGYHGEEVVVIDDFYGWLPWDDLLRLCDRYPLTVETK    240
Po/PCV2/DOM/P4/2021         SKWAANFADPETTYWKPPRNKWWDGYHGEEVVVIDDFYGWLPWDDLLRLCDRYPLTVETK    240
Po/PCV2/DOM/Z11/2021        SKWAANFADPETTYWKPPRNKWWDGYHGEEVVVIDDFYGWLPWDDLLRLCDRYPLTVETK    240
Po/PCV2/DOM/Z13/2021        SKWAANFADPETTYWKPPRNKWWDGYHGEEVVVIDDFYGWLPWDDLLRLCDRYPLTVETK    240
Po/PCV2a/HQ202949           SKWAANFADPETTYWKPPRNKWWDGYHGEEVVVIDDFYGWLPWDDLLRLCDRYPLTVETK    240
Po/PCV2b/DQ220736           SKWAANFADPETTYWKPPRNKWWDGYHGEEVVVIDDFYGWLPWDDLLRLCDRYPLTVETK    240
Po/PCV2c/EU148503           SKWAANFADPETTYWKPPRNKWWDGYHGEEVVVIDDFYGWLPWDDLLRLCDRYPLTVETK    240
Po/PCV2d/MH323413           SKWAANFADPETTYWKPPRNKWWDGYHGEEVVVIDDFYGWLPWDDLLRLCDRYPLTVETK    240
Po/PCV2d/JX535296           SKWAANFADPETTYWKPPRNKWWDGYHGEEVVVIDDFYGWLPWDDLLRLCDRYPLTVETK    240
Po/PCV2d/MF616427           SKWAANFADPETTYWKPPRNKWWDGYHGEEVVVIDDFYGWLPWDDLLRLCDRYPLTVETK    240
Po/PCV2d/MF142267           SKWAANFADPETTYWKPPRNKWWDGYHGEEVVVIDDFYGWLPWDDLLRLCDRYPLTVETK    240
Po/PCV2d/KX831483           SKWAANFADPETTYWKPPRNKWWDGYHGEEVVVIDDFYGWLPWDDLLRLCDRYPLTVETK    240
Po/PCV2e/KT870147           SKWAANFADPETTYWKPPRNKWWDGYHGEEVVVIDDFYGWLPWDDLLRLCDRYPLTVETK    240
Po/PCV2f/LC008137           SKWAANFADPETTYWKPPRNKWWDGYHGEEVVVIDDFYGWLPWDDLLRLCDRYPLTVETK    240
Po/PCV2g/FJ998185           SKWAANFADPETTYWKPPRNKWWDGYHGEEVVVIDDFYGWLPWDDLLRLCDRYPLTVETK    240
Po/PCV2h/JX506730           SKWAANFADPETTYWKPPRNKWWDGYHGEEVVVIDDFYGWLPWDDLLRLCDRYPLTVETK    240
                            *************************************** ***********  ***** *
```

```
Po/PCV2/DOM/DE7/2020        GGTVPFLARSILITSNQTPLEWYSSTAVPAVEALYRRITSLVFWKNATEQSTEEGGQFVT       300
Po/PCV2/DOM/VE22/2020       GGTVPFLARSILITSNQTPLEWYSSTAVPAVEALYRRITSLVFWKNATEQSTEEGGQFVT       300
Po/PCV2/DOM/GES15/2020      GGTVPFLARSILITSNQTPLEWYSSTAVPAVEALYRRITSLVFWKNATEQSTEEGGQFVT       300
Po/PCV2/DOM/ENG22/2020      GGTVPFLARSILITSNQTPLEWYSSTAVPAVEALYRRITSLVFWKNATEQSTEEGGQFVT       300
Po/PCV2/DOM/DE92/2020       GGTVPFLARSILITSNQTPLEWYSSTAVPAVEALYRRITSLVFWKNATEQSTEEGGQFVT       300
Po/PCV2/DOM/DE102/2020      GGTVPFLARSILITSNQTPLEWYSSTAVPAVEALYWRITSLVFWKNATEQSTEEGGQFVT       300
Po/PCV2/DOM/ENG52/2020      GGTVPFLARSILITSNQTPLEWYSSTAVPAVEALYRRITSLVFWKNATEQSTEEGGQFVT       300
Po/PCV2/DOM/GE2/2020        GGTVPFLARSILITSNQTPLEWYSSTAVPAVEALYRRITSLVFWKNATEQSTEEGGQFVT       300
Po/PCV2/DOM/GES7/2020       GGTVPFLARSILITSNQTPLEWYSSTAVPAVEALYRRITSLVFWKNATEQSTEEGGQFVT       300
Po/PCV2/DOM/MA5/2020        GGTVPVLARSILITSNQTPLEWYSSTAVPAVEALYRRITSLVFWKNATEQSTEEGGQFVT       300
Po/PCV2/DOM/M8/2020         GGTVPFLARSILITSNQTPLEWYSSTAVPAVEALYRRITSLVFWKNATEQSTEEGGQFVT       300
Po/PCV2/DOM/MA9/2020        GGTVPFLARSILITSNQTPLEWYSSTAVPAVEALYRRITSLVFWKNATEQSTEEGGQFVT       300
Po/PCV2/DOM/N8/2020         GGTVPFLARSILITSNQTPLEWYSSTAVPAVEALYRRITSLVFWKNATEQSTEEGGQFVT       300
Po/PCV2/DOM/ENG5/2020       GGTVPFLARSILITSNQTPLEWYSSTAVPAVEALYRRITSLVFWKNATEQSTEEGGQFVT       300
Po/PCV2/DOM/P4/2021         GGTVPFLARSILITSNQTPLEWYSSTAVPAVEALYRRITSLVFWKNATEQSTEEGGQFVT       300
Po/PCV2/DOM/Z11/2021        GGTVPFLARSILITSNQTPLEWYSSTAVPAVEALYRRITSLVFWKNATEQSTEEGGQFVT       300
Po/PCV2/DOM/Z13/2021        GGTVPFLARSILITSNQTPLEWYSSTAVPAVEALYRRITSLVFWKNATEQSTEEGGQFVT       300
Po/PCV2a/HQ202949           GGTVPFLARSILITSNQTPLEWYSSTAVPAVEALYRRITSLVFWKNATEQSTEEGGQFVT       300
Po/PCV2b/DQ220736           GGTVPFLARSILITSNQTPLEWYSSTAVPAVEALYRRITSLVFWKNATEQSTEEGGQFVT       300
Po/PCV2c/EU148503           GGTVPFLARSILITSNQTPLEWYSSTAVPAVEALYRRITSLVFWKTATEQSTEEGGQFVT       300
Po/PCV2d/MH323413           GGTVPFLARSILITSNQTPLEWYSSTAVPAVEALYRRITSLVFWKNATEQSTEEGGQFVT       300
Po/PCV2d/JX535296           GGTVPFLARSILITSNQTPLEWYSSTAVPAVEALYRRITSLVFWKNATEQSTEEGGQFVT       300
Po/PCV2d/MF616427           GGTVPFLARSILITSNQTPLEWYSSTAVPAVEALYRRITSLVFWKNATEQSTEEGGQFVT       300
Po/PCV2d/MF142267           GGTVPFLARSILITSNQTPLEWYSSTAVPAVEALYRRITSLVFWKNATEQSTEEGGQFVT       300
Po/PCV2d/KX831483           GGTVPFLARSILITSNQTPLEWYSSTAVPAVEALYRRITSLVFWKNATEQSTEEGGQFVT       300
Po/PCV2e/KT870147           GGTVPFLARSILITSNQTPLEWYSSTAVPAVEALYRRITSLVFWKNATEQSTEEGGQFVT       300
Po/PCV2f/LC008137           GGTVPFLARSILITSNQTPLEWYSSTAVPAVEALYRRITSLVFWKNATEQSTEEGGQFVT       300
Po/PCV2g/FJ998185           GGTVPFLARSILITSNQTPLEWYSSTAVPAVEALYRRITSLVFWKNATEQSTEEGGQFVT       300
Po/PCV2h/JX506730           GGTVPFLARSILITSNQTPLEWYSSTAVPAVEALYRRITSLVFWKNATEQSTEEGGQFVT       300
                            ***** ***************************** ********* **************

Po/PCV2/DOM/DE7/2020        LSPPCPEFPYEINY     314
Po/PCV2/DOM/VE22/2020       LSPPCPEFPYEINY     314
Po/PCV2/DOM/GES15/2020      LSPPCPEFPYEINY     314
Po/PCV2/DOM/ENG22/2020      LSPPCPEFPYEINY     314
Po/PCV2/DOM/DE92/2020       LSPPCPEFPYEINY     314
Po/PCV2/DOM/DE102/2020      LSPPCPEFPYEINY     314
Po/PCV2/DOM/ENG52/2020      LSPPCPEFPYEINY     314
Po/PCV2/DOM/GE2/2020        LSPPCPEFPYEINY     314
Po/PCV2/DOM/GES7/2020       LSPPCPEFPYEINY     314
Po/PCV2/DOM/MA5/2020        LSPPCPEFPYEINY     314
Po/PCV2/DOM/M8/2020         LSPPCPEFPYEINY     314
Po/PCV2/DOM/MA9/2020        LSPPCPEFPYEINY     314
Po/PCV2/DOM/N8/2020         LSPPCPEFPYEINY     314
Po/PCV2/DOM/ENG5/2020       LSPPCPEFPYEINY     314
Po/PCV2/DOM/P4/2021         LSPPCPEFPYEINY     314
Po/PCV2/DOM/Z11/2021        LSPPCPEFPYEINY     314
Po/PCV2/DOM/Z13/2021        LSPPCPEFPYEINY     314
Po/PCV2a/HQ202949           LSPPCPEFPYEINY     314
Po/PCV2b/DQ220736           LSPPCPEFPYEINY     314
Po/PCV2c/EU148503           LSPPCPEFPYEINY     314
Po/PCV2d/MH323413           LSPPCPEFPYEINY     314
Po/PCV2d/JX535296           LSPPCPEFPYEINY     314
Po/PCV2d/MF616427           LSPPCPEFPYEINY     314
Po/PCV2d/MF142267           LSPPCPEFPYEINY     314
Po/PCV2d/KX831483           LSPPCPEFPYEINY     314
Po/PCV2e/KT870147           LSPPCPEFPYEINY     314
Po/PCV2f/LC008137           LSPPRPEFPYEINY     314
Po/PCV2g/FJ998185           LSPPCPEFPYEINY     314
Po/PCV2h/JX506730           LSPPCPEFPYEINY     314
                            **** *********
```
