# Supplementary material for: Detection and Complete Genome Analysis of Porcine Circovirus 2 (PCV2) and an Unclassified CRESS DNA Virus from Diarrheic Pigs in the Dominican Republic: First Evidence for Predominance of PCV2d from the Caribbean Region
Source: Viruses. 2022 Aug 17;14(8):1799. doi: 10.3390/v14081799 (PMC9415081; doi:10.3390/v14081799)
Supplement: Supplementary file 1 [file viruses-14-01799-s001.zip › Supplementary material S7.pdf]

Supplementary material S7. Multiple alignment of the putative replication-associated proteins (Rep) of porcine-associated CRESS DNA virus CRESSV2/ENG22, other porcine-associated CRESS DNA viruses (CRESS group/virus name/GenBank accession number), and porcine circovirus 2 strains (PCV2 genotype/virus name/GenBank accession number). The conserved rolling circle replication (motifs I through III) and superfamily 3 helicase (Walker A and B, and motif C) motifs are shown with green. Sample ENG22 also tested positive for porcine circovirus 2d. A '*' denotes an identical amino acid (aa) residue, whilst '-' indicates absence of an aa residue. Numbers to the right indicate the positions of the aa for respective virus strains.

```
CRESSV2/ENG22               -------MSQRVRATRWVFTINNFTQEDEEAVLNLPYQDWCKSVIAEEEHLDNGTPHIQG       53
CRESSV2/303_7/MW847281      -------MSQRVRATRWVFTINNFTQEDEEAVINLPYQDWCKSVIAEEEHLDDGTPHIQG       53
CRESSV2/453_7/MW847282      -------MSQRVRATRWVFTINNFTQEDEEAVINLPYQDWCKSVIAEEEHLDDGTPHIQG       53
CRESSV2/51/JF713719         ------MSSKRVIATRWVFTINNFTQEDEEAVINLPDQDWCKSVIAEEEHLEDGTPHIQG       54
PCV2d/ENG22                 MPSKKNGRSGPQPHKRWVFTLNNPSEDERKKIRELPISLFDYFIVGEEGNEEGRTPHLQG       60
PCV2d/KU-1503/KX828227      MPSKKSGRSGPQPHKRWVFTLNNPSEDERKKIRELPISLFDYFIVGEEGNEEGRTPHLQG       60
PCV2a/TY1/HQ202949          MPSKKNGRSGPQPHKRWVFTLNNPSEDERKKIRELPISLFDYFIVGEEGNEEGRTPHLQG       60
PCV2b/K-TB60/MN935183       MPSKKNGRSGPQPHKRWVFTLNNPSEDERKKIRDLPISLFDYFIVGEEGNEEGRTPHLQG       60
                                    *      ***** **           **          **      *** **

CRESSV2/ENG22               YMVTDRKDR-SVISRYLNSHAYIEVARGSSYDNWEYCSKEDQIIVEYNKPSEADNKPHRS       112
CRESSV2/303_7/MW847281      YMVTDRKDR-SVISRYLNSHAYIEVARGSSFDNWEYCSKEDQIIVEYNKPSEADNKPHRS       112
CRESSV2/453_7/MW847282      YMVTDRKDR-SVISRYLNSHAYIEVARGSSFDNWEYCSKEDQIIVEYNKPSEADNKPHRS       112
CRESSV2/51/JF713719         YMVTSRKDR-SAISKYLNSHAYIEVARGSSYDNWEYCSKEDQIIVEYNKPSQADNKPHRS       113
PCV2d/ENG22                 FANFVKKQTFNKVKWYLGARCHIEKAKGTDQQNKEYCSKEGNLLIECGAPRSQGQRSDLS       120
PCV2d/KU-1503/KX828227      FANFVKKQTFNKVKWYFGARCHIEKAKGTDQQNKEYCSKEGNLLIECGAPRSQGQRSDLS       120
PCV2a/TY1/HQ202949          FANFVKKQTFNKVKWYLGARCHIEKAKGTDQQNKEYCSKEGNLLIECGAPRSQGQRSDLS       120
PCV2b/K-TB60/MN935183       FANFVKKQTFNKVKWYLGARCHIEKAKGTDQQNKEYCSKEGNLLIECGAPRSQGQRSDLS       120
                                  *        *      ** * *    * ******     *   *         *

CRESSV2/ENG22               LTRNQEESLSFLEACKTYTRDQIIEEFPCLYTRHYQLYEKLHSEYAIRSIDTYDGRLHDK       172
CRESSV2/303_7/MW847281      LTRNQEESLSFLEACKTYTRDQIIEEFPCLYTRHYQLYEKLHSEYAIRSIDTYDGRLHDK       172
CRESSV2/453_7/MW847282      LTRNQEESLSFLEACKTYTRDQIIEEFPCLYTRHYQLYEKLHSEYAIRSIDTYDGRLHDK       172
CRESSV2/51/JF713719         LTRNQEETLSFLESCKTFTRDQMIEQYPCLYARHYQLYEKLHSEYAIRSIDTYDGRLHDK       173
PCV2d/ENG22                 TAV-----STLLESGSL---VTVAEQHPVTFVRNFRGLAELLKVSGKM----QKRDWKTN       168
PCV2d/KU-1503/KX828227      TAV-----STLLESGSL---VTVAEQHPVTFVRNFRGLAELLKVSGKM----QKRDWKTN       168
PCV2a/TY1/HQ202949          TAV-----STLLESGSL---VTVAEQHPVTFVRNFRGLAELLKVSGKM----QKRDWKTN       168
PCV2b/K-TB60/MN935183       TAV-----STLLESGSL---VTVAEQHPVTFVRNFRGLAELLKVSGKM----QKRDWKTN       168
                                       **           *  *    *       *

CRESSV2/ENG22               NLWIYGPAGSGKTTLALSDIEHYK-VYLKPINKWWDGFNPNYHERILIDDFPEGATASIL       231
CRESSV2/303_7/MW847281      NLWIYGPAGSGKTTLALSDIEHYK-VYLKPINKWWDGFNPNYHERILIDDFPEGATASIL       231
CRESSV2/453_7/MW847282      NLWIYGPAGSGKTTLALSDIEHYK-VYLKPINKWWDGFNPNYHERILIDDFPEGATASIL       231
CRESSV2/51/JF713719         NLWIYGPAGSGKTTLALSDIEHYK-VYLKPINKWWDGFNPNYHERILIDDFPEGATASIL       232
PCV2d/ENG22                 VHVIVGPPGCGKSKWAANFADPETTYWKPPRNKWWDGYHGE--EVVVIDDFYGWLPWDD-       225
PCV2d/KU-1503/KX828227      VHVIVGPPGCGKSKWAANFADPETTYWKPPRNKWWDGYHGE--EVVVIDDFYGWLPWDD-       225
PCV2a/TY1/HQ202949          VHVIVGPPGCGKSKWAANFADPETTYWKPPRNKWWDGYHGE--EVVVIDDFYGWLPWDD-       225
PCV2b/K-TB60/MN935183       VHVIVGPPGCGKSKWAANFADPETTYWKPPRNKWWDGYHGE--EVVVIDDFYGWLPWDD-       225
                               * ** * **   *             * ******      *   ****

CRESSV2/ENG22               APFVKHWGDRFPYNAEVKNGSTVINPSIPVIITSNYSIDETFDRQQ--DRDAIRRRFQE-       288
CRESSV2/303_7/MW847281      APFVKHWGDRFPYNAEVKNGSTVINPSIPVIITSNYSIDETFDRQQ--DRDAIRRRFQE-       288
CRESSV2/453_7/MW847282      APFVKHWGDRFPYNAEVKNGSTVINPSIPVIITSNYSIDETFDRQQ--DRDAIRRRFQE-       288
CRESSV2/51/JF713719         APFVKHWGDRFPYNAEVKNGSTVINPSIPVIITSNYSIDETFDRQQ--DRDAVRRRFQE-       289
PCV2d/ENG22                 ---LLRLCDRYPLTVETKGGTVPFLAR-SILITSNQTPLEWYSSTAVPAVEALYRRITSL       281
PCV2d/KU-1503/KX828227      ---LLRLCDRYPLTVETKGGTVPFLAR-SILITSNQTPLEWYSSTAVPAVEALYRRITSL       281
PCV2a/TY1/HQ202949          ---LLRLCDRYPLTVETKGGTVPFLAR-SILITSNQTPLEWYSSTAVPAVEALYRRITSL       281
PCV2b/K-TB60/MN935183       ---LLRLCDRYPLTVETKGGTVPFLAR-SILITSNQTPLEWYSSTAVPAVEALYRRITSL       281
                                    ** *   * * *           ****    *           *  **

CRESSV2/ENG22               -----------------IYLDGSQSHLPYYCHLDLLYSRIRSGEIDKE 319
CRESSV2/303_7/MW847281      -----------------IYLDGSQSHLPYYCHLDLLYSRLRSGEIDK- 318
CRESSV2/453_7/MW847282      -----------------IYLDGSQSHLPYYCHLDLLYSRLRSGEIDKE 319
CRESSV2/51/JF713719         -----------------IYLDGSQSHLPYYCHLDLLYSRIRSGEINKE 320
PCV2d/ENG22                 VFWKNATEQSTEEGGQFVTLSPPCPEFPYEINY--------------- 314
PCV2d/KU-1503/KX828227      VFWKNATEQSTEEGGQFVTLSPPCPEFPYEINY--------------- 314
PCV2a/TY1/HQ202949          VFWKNATEQSTEEGGQFVTLSPPCPEFPYEINY--------------- 314
PCV2b/K-TB60/MN935183       VFWKNATEQSTEEGGQFVTLSPPCPEFPYEINY--------------- 314
                                               *       **
```
