# Supplementary material for: Detection and Complete Genome Analysis of Porcine Circovirus 2 (PCV2) and an Unclassified CRESS DNA Virus from Diarrheic Pigs in the Dominican Republic: First Evidence for Predominance of PCV2d from the Caribbean Region
Source: Viruses. 2022 Aug 17;14(8):1799. doi: 10.3390/v14081799 (PMC9415081; doi:10.3390/v14081799)
Supplement: Supplementary file 1 [file viruses-14-01799-s001.zip › Supplementary material S8.pdf]

Supplementary material S8. Multiple alignment of the putative protein encoded by open reading frame 1 (ORF1) of porcine-associated CRESS DNA virus CRESSV2/ENG22 and other porcine-associated CRESS DNA viruses (virus name/GenBank accession number). Based on the presence of slightly more arginine residues in the amino terminus, which is characteristic of circoviral proteins, it has been speculated that ORF1 of the porcine-associated CRESS DNA viruses might encode the putative capsid protein. A '*' denotes an identical amino acid (aa) residue, whilst '-' indicates absence of an aa residue. Numbers to the right indicate the positions of the aa for respective sequences.

```
CRESSV2/ENG22            MYGRRSYRSPRRSYARAVKPVKYSNETFNASFAYVWATTPTTSMVTMIPAVESLGMRKVK 60
303_7/MW847281           MYGRRSYRSPRRSYARAVKPVKYSNETFNASFAYVWATTPTTSMVTMIPAVESLGMRKVK 60
453_7/MW847282           MYGRRSYRSPRRSYARAVKPVKYSNETFNASFAYVWATTPTTSMVTMIPAVESLGMRKVK 60
PCV-like 51/JF713719     MYGRRSYR-PRRSYARAVKPVKYSNETFNAAFAYVWASTPTTSMVTMIPAVESLGMRKVK 59
                         ******** ********************* ****** **********************

CRESSV2/ENG22            NFTLSITQSPTLDSSNTVKSASSFMYALVYLPDGVTANTLSIGNSTAASLYEPNQNVIAS 120
303_7/MW847281           NFTLSITQSPTLDSSNTVKAASSFMYALVYLPDGVTANTLSIGNSTAASLYEPNQNVIAS 120
453_7/MW847282           NFTLSITQSPTLDSSNTVKAASSFMYALVYLPDGVTANTLSIGNSTAASLYEPNQNVIAS 120
PCV-like 51/JF713719     NFTLSITQSPTLDSSSTVKAASSFMYALVYLPDGVTANTLSIGNSTAASLYEPNQNVIAS 119
                         *************** *** ****************************************

CRESSV2/ENG22            GVCSSSNGQFRLSSRLARNLNSGDRVVLLLRPTSSTGTANDRTNISVILNYSITY      175
303_7/MW847281           GVCSSSNGQFRLSSRLARNLNSGDRVVLLLRPTSSTGTANDRTNISVILNYSITY      175
453_7/MW847282           GVCSSSNGQFRLSSRLARNLNSGDRVVLLLRPTSSTGTANDRTNISVILNYSITY      175
PCV-like 51/JF713719     GVCSSNNGQFRLSSRLARNLNSGDRVVLLLRPTASQGTAGDLTNISVVLNYSITY      174
                         ***** *************************** * *** * ***** *******
```
